# Investigating the impact of non-additive genetic effects in the estimation of variance components and genomic predictions for heat tolerance and performance traits in crossbred and purebred pig populations

**DOI:** 10.1186/s12863-023-01174-x

**Published:** 2023-12-13

**Authors:** Letícia Fernanda de Oliveira, Luiz F. Brito, Daniele Botelho Diniz Marques, Delvan Alves da Silva, Paulo Sávio Lopes, Cassiane Gomes dos Santos, Jay S. Johnson, Renata Veroneze

**Affiliations:** 1 Department of Animal Science, Federal University of Viçosa, Viçosa, MG Brazil; 2 Department of Animal Sciences, Purdue University, West Lafayette, IN USA; 3 USDA-ARS Livestock Behavior Research Unit, West Lafayette, IN USA

**Keywords:** Climatic resilience, Dominance, Epistasis, Landrace, Large White, Maternal-line pigs

## Abstract

**Background:**

Non-additive genetic effects are often ignored in livestock genetic evaluations. However, fitting them in the models could improve the accuracy of genomic breeding values. Furthermore, non-additive genetic effects contribute to heterosis, which could be optimized through mating designs. Traits related to fitness and adaptation, such as heat tolerance, tend to be more influenced by non-additive genetic effects. In this context, the primary objectives of this study were to estimate variance components and assess the predictive performance of genomic prediction of breeding values based on alternative models and two independent datasets, including performance records from a purebred pig population and heat tolerance indicators recorded in crossbred lactating sows.

**Results:**

Including non-additive genetic effects when modelling performance traits in purebred pigs had no effect on the residual variance estimates for most of the traits, but lower additive genetic variances were observed, especially when additive-by-additive epistasis was included in the models. Furthermore, including non-additive genetic effects did not improve the prediction accuracy of genomic breeding values, but there was animal re-ranking across the models. For the heat tolerance indicators recorded in a crossbred population, most traits had small non-additive genetic variance with large standard error estimates. Nevertheless, panting score and hair density presented substantial additive-by-additive epistatic variance. Panting score had an epistatic variance estimate of 0.1379, which accounted for 82.22% of the total genetic variance. For hair density, the epistatic variance estimates ranged from 0.1745 to 0.1845, which represent 64.95–69.59% of the total genetic variance.

**Conclusions:**

Including non-additive genetic effects in the models did not improve the accuracy of genomic breeding values for performance traits in purebred pigs, but there was substantial re-ranking of selection candidates depending on the model fitted. Except for panting score and hair density, low non-additive genetic variance estimates were observed for heat tolerance indicators in crossbred pigs.

**Supplementary Information:**

The online version contains supplementary material available at 10.1186/s12863-023-01174-x.

## Background

 Phenotypes are the result of the individual genotypic value, environmental deviation, and interactions between genotype and the environment. The genotypic values can be divided in additive (breeding values) and non-additive genetic effects due to interactions between alleles at the same locus (dominance) and two or more loci (epistasis) [1]. Non-additive genetic effects are often ignored in livestock genetic evaluations due to the greater importance of additive effects for breeding value estimation, and the fact that breeding animals pass alleles and not genotypes to their offspring [2–5]. Furthermore, non-additive genetic effects can be confounded with non-genetic effects, such as maternal and permanent environmental effects, and the analyses incorporating non-additive genetic effects are often more complex and computationally demanding [4–6].

Although breeders are mainly interested in the estimates of breeding values, fitting non-additive genetic effects in the genomic prediction models can increase the accuracy of the breeding values [7–9]. Furthermore, fitting non-additive genetic effects in the models can yield more accurate predictions of future performance and better mating plans to maximize offspring performance by exploring the combination of additive and non-additive genetic effects [2]. The increasing availability of large-scale genomic datasets has enabled the study of the non-additive actions of genes in numerous complex traits, including the evaluation of non-additive genetic effects in genomic prediction of breeding values (e.g., [2, 9, 10]), genome-wide association studies (e.g., [11–13]), and the development of new methods for the estimation and applications of non-additive genetic effects in breeding programs [6, 9, 14–16]. It is also important to note that accounting for inbreeding in variance component estimation and genomic prediction ensures unbiased variance components, minimizes bias issues in the estimates, accounts for directional dominance, and prevents inflation of dominance variance estimates [17–19].

Non-additive genetic effects are expected to have a greater impact in crossbred performance as compared to purebred populations, mainly due to heterosis [20–22]. As pig production is based on crossbreeding schemes, it is important to evaluate the magnitude of non-additive effects for different traits measured on purebred and crossbred populations, as well as evaluate the impact of these effects on the predictive ability of genomic breeding values. In general, adaptation and fitness traits tend to be more influenced by non-additive genetic effects (e.g., [17, 22–24]). In this context, heat tolerance indicators are key fitness traits to be included in swine breeding programs for improving pig health, welfare, and production [25, 26]. Breeding for improved heat tolerance is becoming more important due to the increase in global temperatures that directly impact swine health, performance, and welfare [27, 28]. Although estimates of additive genetic variance and heritability have been reported for heat stress traits in pigs (e.g., [29–31]), the impact of non-additive effects on direct indicators of heat tolerance in crossbred pigs is still unknown.

Non-additive genetic effects can be of different magnitude in purebred and crossbred populations [20–22], as well for across traits with different genetic architecture. Therefore, there is a need to evaluate the impact of non-additive genetic effects into genomic prediction models for a diverse set of traits, including production, fitness, and adaptation, within both purebred and crossbred populations. In this context, the primary objectives of this study were to estimate additive and non-additive genetic variance components for different trait groups (i.e., production, fitness, and adaptation traits) in independent purebred and crossbred populations. In addition, the impact of non-additive effects in the accuracy of genomic prediction of purebred animals was evaluated.

## Results

The results obtained are presented separately for each of the two datasets evaluated.

### Dataset 1: purebred pig population

#### Variance components and heritability estimates

Variance components and heritabilities were estimated for five traits representing phenotypes routinely collected from birth to breeding in a single nucleus pig line population [32]. Table 1 presents the estimates of variance components and Table 2 shows the estimates of heritability and non-additive genetic variance ratios for all five traits in the purebred pig population. All the fitted models converged for all traits, with the exception of the model including all non-additive genetic effects evaluated and inbreeding depression (MAIDE3) that did not converge for one of the traits (T3). When inbreeding was included as a covariate in the models, a small decrease in the additive genetic variance was observed (Table 1). For most of the traits, lower residual and additive genetic variance estimates were observed when incorporating non-additive genetic effects in the analyses.

**Table 1:** Variance component estimates based on models including or not inbreeding and non-additive genetic effects

Trait	Model^a^	$\widehat{{\sigma }_{a}^{2}}$	$\widehat{{\sigma }_{d}^{2}}$	$\widehat{{\sigma }_{aa}^{2}}$	$\widehat{{\sigma }_{ad}^{2}}$	$\widehat{{\sigma }_{dd}^{2}}$	$\widehat{{\sigma }_{e}^{2}}$
T1	MA	0.0477 ± 0.0214	-	-	-	-	1.4103 ± 0.0418
	MAI	0.0463 ± 0.0214	-	-	-	-	1.4117 ± 0.0418
	MAE	0.0190 ± 0.0229	-	0.1864 ± 0.0942	-	-	1.2540 ± 0.0871
	MAIE	0.0169 ± 0.0229	-	0.1880 ± 0.0945	-	-	1.2545 ± 0.0871
	MAD	0.0477 ± 0.0214	8.92 × 10^−8^ ± 0.0000	-	-	-	1.4103 ± 0.0418
	MAID	0.0463 ± 0.0214	8.93 × 10^−8^ ± 0.0000	-	-	-	1.4117 ± 0.0418
	MADE1	0.0190 ± 0.0229	8.42 × 10^−8^ ± 0.0000	0.1864 ± 0.0956	-	-	1.2542 ± 0.0890
	MAIDE1	0.0169 ± 0.0229	9.89 × 10^−8^ ± 0.0000	0.1880 ± 0.0959	-	-	1.2547 ± 0.0890
	MADE2	0.0190 ± 0.0229	9.10 × 10^−8^ ± 0.0000	0.1864 ± 0.0942	9.96 × 10^−7^ ± 0.0000	-	1.2542 ± 0.0870
	MAIDE2	0.0169 ± 0.0229	9.11 × 10^−8^ ± 0.0000	0.1880 ± 0.0945	1.03 × 10^−6^ ± 0.0000	-	1.2547 ± 0.0870
	MADE3	0.0190 ± 0.0232	8.54 × 10^−8^ ± 0.0000	0.1864 ± 0.1030	4.67 × 10^−7^ ± 0.0000	1.16 × 10^−6^ ± 0.0000	1.2542 ± 0.2248
	MAIDE3	0.0169 ± 0.0232	8.45 × 10^−8^ ± 0.0000	0.1880 ± 0.1033	4.33 × 10^−7^ ± 0.0000	1.22 × 10^−6^ ± 0.0000	1.2547 ± 0.2249
T2	MA	0.2945 ± 0.0339	-	-	-	-	0.7975 ± 0.0277
	MAI	0.2946 ± 0.0340	-	-	-	-	0.7978 ± 0.0277
	MAE	0.2276 ± 0.0355	-	0.2643 ± 0.0766	-	-	0.5943 ± 0.0617
	MAIE	0.2263 ± 0.0356	-	0.2665 ± 0.0768	-	-	0.5934 ± 0.0618
	MAD	0.2927 ± 0.0339	0.0221 ± 0.0240	-	-	-	0.7766 ± 0.0350
	MAID	0.2926 ± 0.0340	0.0224 ± 0.0238	-	-	-	0.7767 ± 0.0350
	MADE1	0.2266 ± 0.0354	0.0201 ± 0.0237	0.2625 ± 0.0768	-	-	0.5768 ± 0.0639
	MAIDE1	0.2250 ± 0.0355	0.0209 ± 0.0238	0.2651 ± 0.0768	-	-	0.5750 ± 0.0640
	MADE2	0.2267 ± 0.0354	0.0201 ± 0.0237	0.2625 ± 0.0768	9.23 × 10^−7^ ± 0.0000	-	0.5768 ± 0.0639
	MAIDE2	0.2250 ± 0.0355	0.0209 ± 0.0238	0.2651 ± 0.0768	9.20 × 10^−7^ ± 0.0000	-	0.5750 ± 0.0640
	MADE3	0.2267 ± 0.0354	0.0201 ± 0.0237	0.2625 ± 0.0768	9.23 × 10^−7^ ± 0.0000	1.20 × 10^−6^ ± 0.0000	0.5768 ± 0.0639
	MAIDE3	0.2250 ± 0.0355	0.0209 ± 0.0238	0.2651 ± 0.0768	9.20 × 10^−7^ ± 0.0000	1.20 × 10^−6^ ± 0.0000	0.5750 ± 0.0640
T3	MA	0.1881 ± 0.0257	-	-	-	-	0.7151 ± 0.0227
	MAI	0.1869 ± 0.0256	-	-	-	-	0.7158 ± 0.0227
	MAE	0.1049 ± 0.0239	-	0.3895 ± 0.0625	-	-	0.4094 ± 0.0494
	MAIE	0.1033 ± 0.0238	-	0.3901 ± 0.0625	-	-	0.4097 ± 0.0494
	MAD	0.1857 ± 0.0257	0.0186 ± 0.0207	-	-	-	0.6982 ± 0.0291
	MAID	0.1845 ± 0.0256	0.0194 ± 0.0209	-	-	-	0.6982 ± 0.0292
	MADE1	0.1048 ± 0.0239	1.10 × 10^−7^ ± 0.0000	0.3891 ± 0.0632	-	-	0.4090 ± 0.0506
	MAIDE1	0.1032 ± 0.0238	1.43 × 10^−7^ ± 0.0000	0.3897 ± 0.0632	-	-	0.4093 ± 0.0506
	MADE2	0.1133 ± 0.0243	2.61 × 10^−7^ ± 0.0000	0.2550 ± 0.0704	0.4553 ± 0.1227	-	0.0802 ± 0.0991
	MAIDE2	0.1118 ± 0.0243	3.08 × 10^−7^ ± 0.0000	0.2561 ± 0.0705	0.4536 ± 0.1229	-	0.0818 ± 0.0986
	MADE3	0.1139 ± 0.0242	1.89 × 10^−7^ ± 0.0000	0.2656 ± 0.0695	0.0841 ± 0.2002	0.4381 ± 0.1849	1.19 × 10^−4^ ± 0.0000
	MAIDE3^b^	-	-	-	-	-	-
T4	MA	1.7199 ± 0.1708	-	-	-	-	3.4330 ± 0.1147
	MAI	1.7206 ± 0.1710	-	-	-	-	3.4340 ± 0.1148
	MAE	1.5743 ± 0.1846	-	0.4672 ± 0.2938	-	-	3.0851 ± 0.2441
	MAIE	1.5738 ± 0.1845	-	0.4727 ± 0.2936	-	-	3.0819 ± 0.2444
	MAD	1.7172 ± 0.1710	0.0576 ± 0.0847	-	-	-	3.3777 ± 0.1378
	MAID	1.7178 ± 0.1711	0.0585 ± 0.0848	-	-	-	3.3779 ± 0.1379
	MADE1	1.5794 ± 0.1849	0.0351 ± 0.0835	0.4445 ± 0.2983	-	-	3.0682 ± 0.2476
	MAIDE1	1.5790 ± 0.1851	0.0355 ± 0.0826	0.4494 ± 0.2976	-	-	3.0649 ± 0.2480
	MADE2	1.5794 ± 8.5400	0.0351 ± 0.4200	0.4445 ± 0.2983	3.10 × 10^−7^ ± 0.0000	-	3.0681 ± 0.2476
	MAIDE2	1.5790 ± 0.1851	0.0355 ± 0.0826	0.4494 ± 0.2976	3.10 × 10^−7^ ± 0.0000	-	3.0649 ± 0.2480
	MADE3	1.5794 ± 0.1849	0.0351 ± 0.0835	0.4445 ± 0.2983	4.91 × 10^−7^ ± 0.0000	6.38 × 10^−6^ ± 0.0000	3.0681 ± 0.2476
	MAIDE3	1.5790 ± 0.1851	0.0355 ± 0.0826	0.4494 ± 0.2976	4.90 × 10^−7^ ± 0.0000	6.37 × 10^−6^ ± 0.0000	3.0649 ± 0.2480
T5	MA	1,310.6200 ± 122.0317	-	-	-	-	2,190.1200 ± 74.7226
	MAI	1,313.0200 ± 122.1414	-	-	-	-	2,186.8400 ± 74.6617
	MAE	1,146.0400 ± 129.7894	-	539.2420 ± 202.7226	-	-	1,786.3800 ± 163.5879
	MAIE	1,149.1100 ± 129.8429	-	535.6400 ± 202.8939			1,786.0400 ± 163.7067
	MAD	1,303.6700 ± 121.8383	171.8660 ± 65.5977	-	-	-	2,022.8200 ± 90.7908
	MAID	1,305.2600 ± 121.8730	158.4950 ± 65.4938	-	-	-	2,033.7900 ± 90.9973
	MADE1	1,168.3800 ± 130.5453	151.9660 ± 64.9427	451.3190 ± 205.1450	-	-	1,703.5000 ± 167.0098
	MAIDE1	1,167.8900 ± 130.4905	138.0280 ± 64.4991	457.6490 ± 205.2238	-	-	1,710.3400 ± 167.0254
	MADE2	1,168.3800 ± 130.5453	151.9660 ± 64.9427	451.3180 ± 205.1445	0.0012 ± 0.0000	-	1,703.4900 ± 167.0088
	MAIDE2	1,167.8900 ± 130.4905	138.0280 ± 64.4991	457.6490 ± 205.2238	0.0012 ± 0.0000	-	1,710.3400 ± 167.0254
	MAIDE3	1,167.8800 ± 130.9283	138.0280 ± 65.7276	457.6430 ± 231.1328	0.0012 ± 0.0000	0.0036 ± 0.0000	1,710.3200 ± 337.3412

$\widehat{{\sigma }_{a}^{2}}$: additive genetic variance estimate

$\widehat{{\sigma }_{d}^{2}}$: dominance variance estimate

$\widehat{{\sigma }_{aa}^{2}}$: additive-by-additive epistatic variance estimate

$\widehat{{\sigma }_{ad}^{2}}$: additive-by-dominance epistatic variance estimate

$\widehat{{\sigma }_{dd}^{2}}$: dominance-by-dominance epistatic variance estimate

$\widehat{{\sigma }_{e}^{2}}$: residual variance estimate

^a^MA: $\textbf{y}=\textbf{X}\varvec{\upbeta }+\textbf{Za}+\varvec{\upepsilon }$ ; MAI: $\textbf{y}=\textbf{X}\varvec{\upbeta }+\textbf{fb}+\textbf{Za}+\varvec{\upepsilon }$; MAE: $\textbf{y}=\textbf{X}\varvec{\upbeta}+\textbf{Za}+\textbf{Z}{\varvec{e}}_{\textbf{aa}}+\varvec{\upepsilon }$; MAIE: $\textbf{y}=\textbf{X}\varvec{\upbeta}+\textbf{fb}+\textbf{Za}+\textbf{Z}{\varvec{e}}_{\textbf{aa}}+\varvec{\upepsilon }$; MAD: $\textbf{y}=\textbf{X}\varvec{\upbeta }+\textbf{Za}+\textbf{Zd}+\varvec{\upepsilon }$; MAID: $\textbf{y}=\textbf{X}\varvec{\upbeta }+\textbf{fb}+\textbf{Za}+\textbf{Zd}+\varvec{\upepsilon }$; MADE1: $\textbf{y}=\textbf{X}\varvec{\upbeta }+\textbf{Za}+\textbf{Zd}+\textbf{Z}{\varvec{e}}_{\textbf{aa}}+\varvec{\upepsilon }$; MAIDE1: $\textbf{y}=\textbf{X}\varvec{\upbeta }+\textbf{fb}+\textbf{Za}+\textbf{Zd}+\textbf{Z}{\varvec{e}}_{\textbf{aa}}+\varvec{\upepsilon }$; MADE2: $\textbf{y}=\textbf{X}\varvec{\upbeta }+\textbf{Za}+\textbf{Zd}+\textbf{Z}{\varvec{e}}_{\textbf{aa}}+\textbf{Z}{\varvec{e}}_{\textbf{ad}}+\varvec{\upepsilon }$; MAIDE2: $\textbf{y}=\textbf{X}\varvec{\upbeta }+\textbf{fb}+\textbf{Za}+\textbf{Zd}+\textbf{Z}{\varvec{e}}_{\textbf{aa}}+\textbf{Z}{\varvec{e}}_{\textbf{ad}}+\varvec{\upepsilon }$; MADE3: $\textbf{y}=\textbf{X}\varvec{\upbeta }+\textbf{Za}+\textbf{Zd}+\textbf{Z}{\varvec{e}}_{\textbf{aa}}+\textbf{Z}{\varvec{e}}_{\textbf{ad}}+\textbf{Z}{\varvec{e}}_{\textbf{dd}}+\varvec{\upepsilon }$; MAIDE3:$\textbf{y}=\textbf{X}\varvec{\upbeta }+\textbf{fb}+\textbf{Za}+\textbf{Zd}+\textbf{Z}{\varvec{e}}_{\textbf{aa}}+\textbf{Z}{\varvec{e}}_{\textbf{ad}}+\textbf{Z}{\varvec{e}}_{\textbf{dd}}+\varvec{\upepsilon }$

^b^Model MAIDE3 did not converge for T3.

**Table 2:** Heritability estimates based on models including or not inbreeding and non-additive genetic effects

Trait	Model^a^	${h}_{a}^{2}$	${h}_{d}^{2}$	${h}_{aa}^{2}$	${h}_{ad}^{2}$	${h}_{dd}^{2}$
T1	MA	0.0327 ± 0.0146	-	-	-	-
	MAI	0.0318 ± 0.0146	-	-	-	-
	MAE	0.0130 ± 0.0157	-	0.1277 ± 0.0642	-	-
	MAIE	0.0116 ± 0.0157	-	0.1288 ± 0.0642	-	-
	MAD	0.0327 ± 0.0147	6.12 × 10^−08^ ± 0.0232	-	-	-
	MAID	0.0318 ± 0.0147	6.12 × 10^−08^ ± 0.0232	-	-	-
	MADE1	0.0130 ± 0.0157	5.77 × 10^−08^ ± 0.0000	0.1277 ± 0.0642	-	-
	MAIDE1	0.0116 ± 0.0157	6.77 × 10^−08^ ± 0.0241	0.1288 ± 0.0652	-	-
	MADE2	0.0130 ± 0.0160	6.23 × 10^−08^ ± 0.0247	0.1277 ± 0.0743	6.82 × 10^−07^ ± 0.1535	-
	MAIDE2	0.0116 ± 0.0160	6.23 × 10^−08^ ± 0.0247	0.1288 ± 0.0743	6.82 × 10^−07^ ± 0.1536	-
	MADE3	0.0130 ± 0.0160	5.85 × 10^−08^ ± 0.0249	0.1277 ± 0.0743	3.20 × 10^−07^ ± 0.2644	7.96 × 10^−07^ ± 0.2910
	MAIDE3	0.0116 ± 0.0160	5.79 × 10^−08^ ± 0.0248	0.1288 ± 0.0743	2.97 × 10^−07^ ± 0.2646	8.35 × 10^−07^ ± 0.2913
T2	MA	0.2697 ± 0.0263	-	-	-	-
	MAI	0.2696 ± 0.0264	-	-	-	-
	MAE	0.2095 ± 0.0300	-	0.2433 ± 0.0698	-	-
	MAIE	0.2083 ± 0.0301	-	0.2453 ± 0.0700	-	-
	MAD	0.2680 ± 0.0265	0.0205 ± 0.0219	-	-	-
	MAID	0.2680 ± 0.0265	0.0205 ± 0.0219	-	-	-
	MADE1	0.2087 ± 0.0299	0.0185 ± 0.0217	0.2417 ± 0.0700	-	-
	MAIDE1	0.2071 ± 0.0301	0.0193 ± 0.0219	0.2441 ± 0.0701	-	-
	MADE2	0.2086 ± 0.0301	0.0185 ± 0.0222	0.2417 ± 0.0775	8.50 × 10^−07^ ± 0.1390	-
	MAIDE2	0.2071 ± 0.0302	0.0193 ± 0.0223	0.2441 ± 0.0776	8.47 × 10^−07^ ± 0.1391	-
	MADE3	0.2086 ± 0.0301	0.0185 ± 0.0224	0.2417 ± 0.0775	8.50 × 10^−07^ ± 0.2355	1.10 × 10^−06^ ± 0.2698
	MAIDE3	0.2071 ± 0.0302	0.0193 ± 0.0225	0.2441 ± 0.0776	8.47 × 10^−07^ ± 0.2357	1.10 × 10^−06^ ± 0.2703
T3	MA	0.2083 ± 0.0253	-	-	-	-
	MAI	0.2071 ± 0.0253	-	-	-	-
	MAE	0.1161 ± 0.0255	-	0.4309 ± 0.0663	-	-
	MAIE	0.1144 ± 0.0254	-	0.4319 ± 0.0663	-	-
	MAD	0.2058 ± 0.0254	0.0206 ± 0.0230	-	-	-
	MAID	0.2045 ± 0.0253	0.0215 ± 0.0231	-	-	-
	MADE1	0.1161 ± 0.0255	1.22 × 10^−07^ ± 0.0210	0.4309 ± 0.0669	-	-
	MAIDE1	0.1144 ± 0.0254	1.59 × 10^−07^ ± 0.0211	0.4319 ± 0.0669	-	-
	MADE2	0.1254 ± 0.0258	2.89 × 10^−08^ ± 0.0222	0.2821 ± 0.0769	0.5037 ± 0.1373	-
	MAIDE2	0.1238 ± 0.0257	3.41 × 10^−08^ ± 0.0222	0.2835 ± 0.0769	0.5022 ± 0.1375	-
	MADE3	0.1263 ± 0.0257	2.10 × 10^−10^ ± 0.0216	0.2946 ± 0.0759	0.0932 ± 0.2235	0.4859 ± 0.2068
	MAIDE3^b^	-	-	-	-	-
T4	MA	0.3338 ± 0.0263	-	-	-	-
	MAI	0.3338 ± 0.0263	-	-	-	-
	MAE	0.3071 ± 0.0304	-	0.0911 ± 0.0574	-	-
	MAIE	0.3069 ± 0.0304	-	0.0922 ± 0.0575	-	-
	MAD	0.3333 ± 0.0263	0.0112 ± 0.0164	-	-	-
	MAID	0.3333 ± 0.0263	0.0114 ± 0.0164	-	-	-
	MADE1	0.3080 ± 0.0304	0.0068 ± 0.0161	0.0867 ± 0.0581	-	-
	MAIDE1	0.3079 ± 0.0304	0.0069 ± 0.0162	0.0876 ± 0.0582	-	-
	MADE2	0.3080 ± 0.0306	0.0068 ± 0.0165	0.0867 ± 0.0656	6.06 × 10^−08^ ± 0.1172	-
	MAIDE2	0.3079 ± 0.0306	0.0069 ± 0.0165	0.0876 ± 0.0657	6.05 × 10^−08^ ± 0.1173	-
	MADE3	0.3080 ± 0.0306	0.0068 ± 0.0166	0.0867 ± 0.0656	9.57 × 10^−07^ ± 0.1856	1.24 × 10^−06^ ± 0.2251
	MAIDE3	0.3079 ± 0.0306	0.0069 ± 0.0166	0.0876 ± 0.0657	9.56 × 10^−07^ ± 0.1860	1.24 × 10^−06^ ± 0.2256
T5	MA	0.3744 ± 0.0264	-	-	-	-
	MAI	0.3752 ± 0.0264	-	-	-	-
	MAE	0.3301 ± 0.0309	-	0.1553 ± 0.0584	-	-
	MAIE	0.3311 ± 0.0310	-	0.1543 ± 0.0585	-	-
	MAD	0.3727 ± 0.0265	0.0491 ± 0.0187	-	-	-
	MAID	0.3732 ± 0.0265	0.0453 ± 0.0187	-	-	-
	MADE1	0.3362 ± 0.0309	0.0437 ± 0.0186	0.1299 ± 0.0590	-	-
	MAIDE1	0.3362 ± 0.0309	0.0397 ± 0.0186	0.1317 ± 0.0591	-	-
	MADE2	0.3362 ± 0.0310	0.0437 ± 0.0190	0.1299 ± 0.0666	3.36 × 10^−07^ ± 0.1166	-
	MAIDE2	0.3362 ± 0.0311	0.0397 ± 0.0189	0.1317 ± 0.0667	3.55 × 10^−07^ ± 0.1166	-
	MADE3	0.3362 ± 0.0311	0.0437 ± 0.0190	0.1299 ± 0.0666	3.33 × 10^−07^ ± 0.1860	1.02 × 10^−06^ ± 0.2256
	MAIDE3	0.3362 ± 0.0311	0.0397 ± 0.0190	0.1317 ± 0.0667	3.58 × 10^−07^ ± 0.1862	1.02 × 10^−06^ ± 0.2151

${h}_{a}^{2}$: additive heritability or narrow-sense heritability

${h}_{d}^{2}$: dominance variance ratio

${h}_{aa}^{2}$: epistatic additive-by-additive variance ratio

${h}_{ad}^{2}$: epistatic additive-by-dominance variance ratio

${h}_{dd}^{2}$: epistatic dominance-by-dominance variance ratio

^a^MA: $\textbf{y}=\textbf{X}\varvec{\upbeta }+\textbf{Za}+\varvec{\upepsilon }$; MAI: $\textbf{y}=\textbf{X}\varvec{\upbeta }+\textbf{fb}+\textbf{Za}+\varvec{\upepsilon }$; MAE: $\textbf{y}=\textbf{X}\varvec{\upbeta }+\textbf{Za}+\textbf{Z}{\varvec{e}}_{\textbf{aa}}+\varvec{\upepsilon }$; MAIE: $\textbf{y}=\textbf{X}\varvec{\upbeta }+\textbf{fb}+\textbf{Za}+\textbf{Z}{\varvec{e}}_{\textbf{aa}}+\varvec{\upepsilon }$; MAD: $\textbf{y}=\textbf{X}\varvec{\upbeta }+\textbf{Za}+\textbf{Zd}+\varvec{\upepsilon }$; MAID: $\textbf{y}=\textbf{X}\varvec{\upbeta }+\textbf{fb}+\textbf{Za}+\textbf{Zd}+\varvec{\upepsilon }$; MADE1: $\textbf{y}=\textbf{X}\varvec{\upbeta }+\textbf{Za}+\textbf{Zd}+\textbf{Z}{\varvec{e}}_{\textbf{aa}}+\varvec{\upepsilon }$; MAIDE1: $\textbf{y}=\textbf{X}\varvec{\upbeta }+\textbf{fb}+\textbf{Za}+\textbf{Zd}+\textbf{Z}{\varvec{e}}_{\textbf{aa}}+\varvec{\upepsilon }$; MADE2: $\textbf{y}=\textbf{X}\varvec{\upbeta }+\textbf{Za}+\textbf{Zd}+\textbf{Z}{\varvec{e}}_{\textbf{aa}}+\textbf{Z}{\varvec{e}}_{\textbf{ad}}+\varvec{\upepsilon }$; MAIDE2: $\textbf{y}=\textbf{X}\varvec{\upbeta }+\textbf{fb}+\textbf{Za}+\textbf{Zd}+\textbf{Z}{\varvec{e}}_{\textbf{aa}}+\textbf{Z}{\varvec{e}}_{\textbf{ad}}+\varvec{\upepsilon }$; MADE3: $\textbf{y}=\textbf{X}\varvec{\upbeta }+\textbf{Za}+\textbf{Zd}+\textbf{Z}{\varvec{e}}_{\textbf{aa}}+\textbf{Z}{\varvec{e}}_{\textbf{ad}}+\textbf{Z}{\varvec{e}}_{\textbf{dd}}+\varvec{\upepsilon }$; MAIDE3:$\textbf{y}=\textbf{X}\varvec{\upbeta }+\textbf{fb}+\textbf{Za}+\textbf{Zd}+\textbf{Z}{\varvec{e}}_{\textbf{aa}}+\textbf{Z}{\varvec{e}}_{\textbf{ad}}+\textbf{Z}{\varvec{e}}_{\textbf{dd}}+\varvec{\upepsilon }$

^b^Model MAIDE3 did not converge for T3.

Low dominance variance components were estimated, ranging from 0 to 0.0576 for T1, T2, T3, and T4, and ranging from 138.0280 to 171.8660 for T5. Notably, the dominance variance ratios across all traits were found to be small, accompanied by relatively large standard errors. T5 had the highest dominance variance ratio, which ranged from 0.0397 to 0.0437, while for the other traits it ranged from 0 to 0.0206. However, the estimates of additive-by-additive epistatic variance were notably higher than the dominance variance estimates. These ratios were approximately 0.12 for T1, 0.24 for T2, 0.28 to 0.43 for T3, 0.09 for T4, and 0.13 to 0.15 for T5, indicating a more substantial contribution of additive-by-additive epistasis to the variance components for these traits.

In general, there was a greater decrease in the additive genetic variance when additive-by-additive epistasis was included in the models (MAE, MAIE, MADE1, MAIDE1, MADE2, MAIDE2, MADE3, MAIDE3). However, for T1 the additive genetic variance estimates also presented larger standard errors (Table 1), indicating that the inclusion of epistasis in the model decreased precision of the additive genetic variance estimates for T1. As a consequence of the decrease in the additive genetic variance, a reduction in the heritability estimates (${h}_{a}^{2}$) were observed when additive-by-additive epistasis was fitted in the models (Table 2). Nevertheless, the variance components for additive-by-dominance and dominance-by-dominance epistasis were small and their variance ratios (${h}_{ad}^{2}$ and ${h}_{dd}^{2}$) were close to zero for almost all traits, except T3.

#### Predictive ability of genomic breeding values

**Fig. 1:**
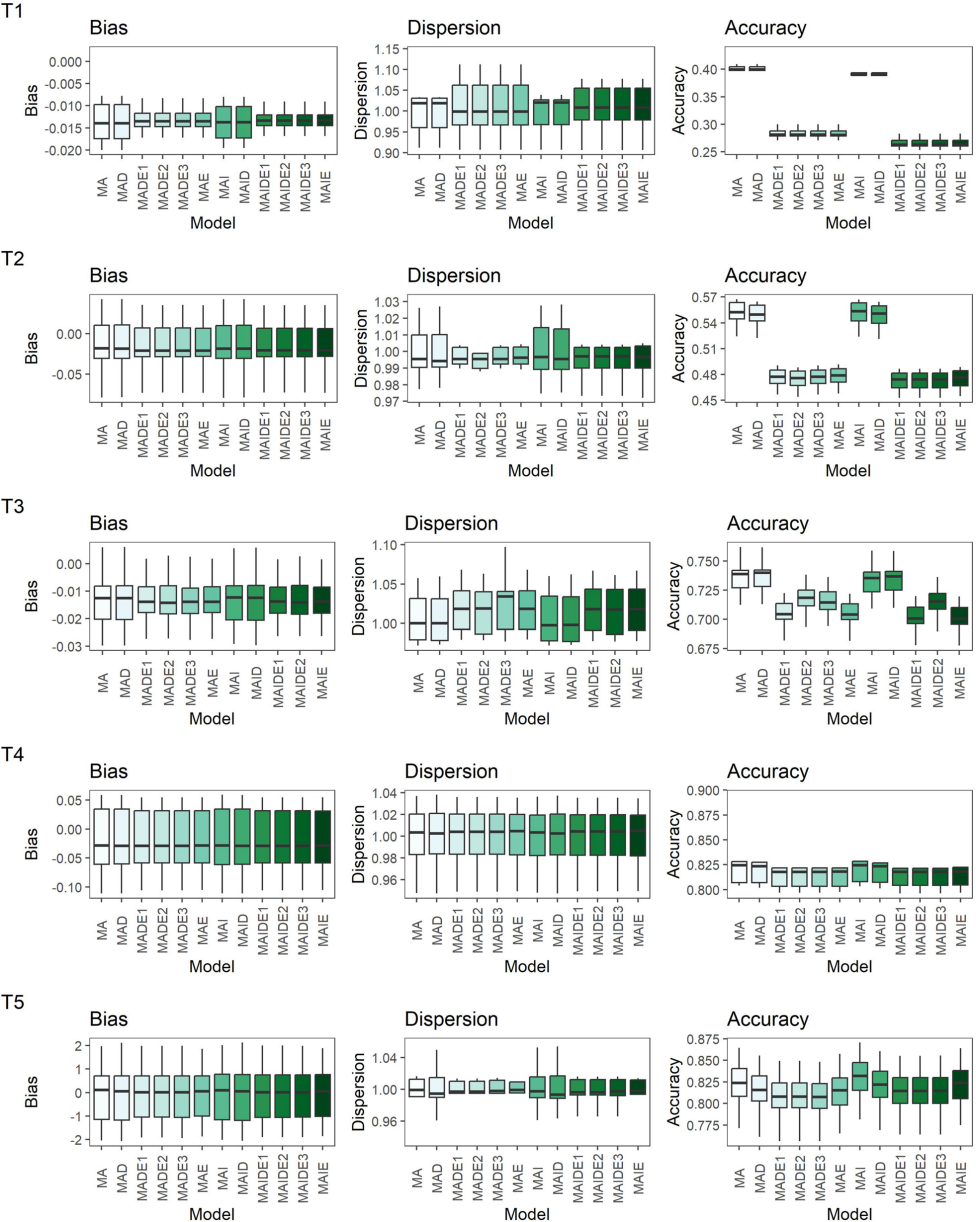
Bias, dispersion, and accuracy estimates of genomic breeding values obtained using the Linear Regression method

 Figure 1 presents the average bias, dispersion, and accuracy values obtained for the genomic breeding values for all five traits. As expected, greater accuracies were obtained for traits with higher ${h}_{a}^{2}$. Fitting inbreeding and/or dominance in the models did not impact the accuracy of breeding values compared to those obtained based on the MA model (Fig. 1). Models including epistasis (MAE, MAIE, MADE1, MAIDE1, MADE2, MAIDE2, MADE3, and MAIDE3) presented on average a relative decrease in accuracy of 31.58%, 13.97%, 3.50%, 0.82%, and 1.34% for T1, T2, T3, T4, and T5, respectively, when compared to MA (Fig. 1). The observed bias was close to zero and the dispersion estimates were close to one for all traits and models (Fig. 1), indicating that the inclusion of non-additive genetic effects in the model did not influence the dispersion of breeding values.

In this section, all models incorporating non-additive genetic effects were compared to the additive animal model. To assess the impact of including non-additive genetic effects in the model on animals’ selection, we calculated the change in an animals’ ranking in comparison to the additive model (MA) based on Spearman’s rank correlation, as shown in Table 3. The percentage of commonly selected individuals when selecting the top 1%, 5%, 10%, and 20% based on different genomic prediction models were compared to the selected individuals based on MA. Table 4 shows the percentage of coincidence of the top 1%, 5%, 10%, and 20% best animals between the MA and the models including non-additive genetic effects. The models including epistasis effect presented the lowest percentage of coincidence of the top animals in comparison to the MA model. This percentage varied from 68.57% for trait T3 to 97.59% for T4. Although there was little change in the Spearman correlations across models (Table 3), the percentage of coincidence between the best animals according to MA were higher for models including inbreeding and/or dominance (MAI, MAD, and MAID). However, the percentage of coincidence decreased for models including epistasis (MAE, MAIE, MADE1, MAIDE1, MADE2, MAIDE2, MADE3, and MAIDE3), as shown in Table 4.

**Table 3:** Spearman’s rank correlation between the additive genetic model (MA) and other models considering non-additive genetic effects

Model^a^	T1	T2	T3	T4	T5
MAI	0.9990	1.000	0.9996	1.0000	0.9993
MAE	0.9844	0.9952	0.9645	0.9989	0.9968
MAIE	0.9856	0.9952	0.9630	0.9989	0.996
MAD	1.0000	0.9999	0.9998	0.9999	0.9985
MAID	0.9990	0.9999	0.9995	0.9999	0.9985
MADE1	0.9844	0.9951	0.9644	0.9989	0.9966
MAIDE1	0.9856	0.9950	0.9630	0.9989	0.9964
MADE2	0.9844	0.9951	0.9692	0.9989	0.9966
MAIDE2	0.9856	0.9950	0.9678	0.9989	0.9964
MADE3	0.9844	0.9951	0.9716	0.9989	0.9966
MAIDE3^b^	0.9856	0.9950	-	0.9989	0.9964

^a^MA: $\textbf{y}=\textbf{X}\varvec{\upbeta }+\textbf{Za}+\varvec{\upepsilon }$; MAI: $\textbf{y}=\textbf{X}\varvec{\upbeta }+\textbf{fb}+\textbf{Za}+\varvec{\upepsilon }$; MAE: $\textbf{y}=\textbf{X}\varvec{\upbeta }+\textbf{Za}+\textbf{Z}{\varvec{e}}_{\varvec{a}\varvec{a}}+\varvec{\upepsilon }$; MAIE: $\textbf{y}=\textbf{X}\varvec{\upbeta }+\textbf{fb}+\textbf{Za}+\textbf{Z}{\varvec{e}}_{\textbf{aa}}+\varvec{\upepsilon }$; MAD: $\textbf{y}=\textbf{X}\varvec{\upbeta }+\textbf{Za}+\textbf{Zd}+\varvec{\upepsilon }$; MAID: $\textbf{y}=\textbf{X}\varvec{\upbeta }+\textbf{fb}+\textbf{Za}+\textbf{Zd}+\varvec{\upepsilon }$; MADE1: $\textbf{y}=\textbf{X}\varvec{\upbeta }+\textbf{Za}+\textbf{Zd}+\textbf{Z}{\varvec{e}}_{\textbf{aa}}+\varvec{\upepsilon }$; MAIDE1: $\textbf{y}=\textbf{X}\varvec{\upbeta }+\textbf{fb}+\textbf{Za}+\textbf{Zd}+\textbf{Z}{\varvec{e}}_{\textbf{aa}}+\varvec{\upepsilon }$; MADE2: $\textbf{y}=\textbf{X}\varvec{\upbeta }+\textbf{Za}+\textbf{Zd}+\textbf{Z}{\varvec{e}}_{\textbf{aa}}+\textbf{Z}{\varvec{e}}_{\textbf{ad}}+\varvec{\upepsilon }$; MAIDE2: $\textbf{y}=\textbf{X}\varvec{\upbeta }+\textbf{fb}+\textbf{Za}+\textbf{Zd}+\textbf{Z}{\varvec{e}}_{\textbf{aa}}+\textbf{Z}{\varvec{e}}_{\textbf{ad}}+\varvec{\upepsilon }$; MADE3: $\textbf{y}=\textbf{X}\varvec{\upbeta }+\textbf{Za}+\textbf{Zd}+\textbf{Z}{\varvec{e}}_{\textbf{aa}}+\textbf{Z}{\varvec{e}}_{\textbf{ad}}+\textbf{Z}{\varvec{e}}_{\textbf{dd}}+\varvec{\upepsilon }$; MAIDE3:$\textbf{y}=\textbf{X}\varvec{\upbeta }+\textbf{fb}+\textbf{Za}+\textbf{Zd}+\textbf{Z}{\varvec{e}}_{\textbf{aa}}+\textbf{Z}{\varvec{e}}_{\textbf{ad}}+\textbf{Z}{\varvec{e}}_{\textbf{dd}}+\varvec{\upepsilon }$

^b^The model MAIDE3 did not converge for T3

**Table 4:** Percentage of commonly selected animals between the additive model and models including non-additive genetic effects

Trait	Model^a^	1%	5%	10%	20%
T1	MAI	97.14%	96.02%	96.88%	97.73%
	MAE	82.86%	86.93%	88.10%	89.09%
	MAIE	82.86%	86.36%	87.54%	89.38%
	MAD	100.00%	100.00%	100.00%	100.00%
	MAID	97.14%	96.02%	96.88%	97.73%
	MADE1	82.86%	86.36%	88.10%	89.09%
	MAIDE1	82.86%	86.36%	87.25%	89.38%
	MADE2	82.86%	86.36%	88.10%	89.09%
	MAIDE2	82.86%	86.36%	87.25%	89.38%
	MADE3	82.86%	86.36%	88.10%	89.09%
	MAIDE3	82.86%	86.36%	87.25%	89.38%
T2	MAI	100.00%	100.00%	100.00%	100.00%
	MAE	77.14%	92.05%	91.78%	94.05%
	MAIE	77.14%	91.48%	91.78%	94.33%
	MAD	97.14%	98.86%	98.58%	99.15%
	MAID	97.14%	98.30%	98.30%	99.15%
	MADE1	80.00%	92.05%	91.50%	94.05%
	MAIDE1	77.14%	92.05%	91.50%	94.05%
	MADE2	80.00%	92.05%	91.50%	94.05%
	MAIDE2	77.14%	92.05%	91.50%	94.05%
	MADE3	80.00%	92.05%	91.50%	94.05%
	MAIDE3	77.14%	92.05%	91.50%	94.05%
T3	MAI	94.29%	97.73%	98.30%	98.30%
	MAE	71.43%	76.14%	79.32%	84.56%
	MAIE	71.43%	77.27%	78.47%	84.70%
	MAD	94.29%	99.43%	98.87%	99.15%
	MAID	94.29%	97.73%	97.73%	98.16%
	MADE1	71.43%	76.14%	79.32%	84.56%
	MAIDE1	71.43%	77.27%	78.47%	84.70%
	MADE2	71.43%	76.70%	81.59%	86.54%
	MAIDE2	71.43%	78.41%	80.45%	86.40%
	MADE3	68.57%	77.84%	81.87%	87.11%
	MAIDE3^b^	-	-	-	-
T4	MAI	100.00%	100.00%	99.72%	99.72%
	MAE	94.29%	97.73%	96.60%	97.45%
	MAIE	94.29%	97.73%	96.60%	97.59%
	MAD	100.00%	98.86%	99.43%	99.43%
	MAID	100.00%	98.86%	99.15%	99.58%
	MADE1	97.14%	97.73%	96.60%	97.45%
	MAIDE1	97.14%	97.73%	96.60%	97.59%
	MADE2	97.14%	97.73%	96.60%	97.45%
	MAIDE2	97.14%	97.73%	96.60%	97.59%
	MADE3	97.14%	97.73%	96.60%	97.45%
	MAIDE3	97.14%	97.73%	96.60%	97.59%
T5	MAI	100.00%	96.02%	96.32%	98.16%
	MAE	88.57%	95.45%	95.18%	96.03%
	MAIE	88.57%	94.32%	94.05%	95.61%
	MAD	94.29%	96.59%	96.88%	97.45%
	MAID	94.29%	96.59%	96.32%	97.45%
	MADE1	91.43%	94.32%	95.75%	96.03%
	MAIDE1	88.57%	95.45%	94.62%	95.61%
	MADE2	91.43%	94.32%	95.75%	96.03%
	MAIDE2	88.57%	95.45%	94.62%	95.61%
	MADE3	91.43%	94.32%	95.75%	96.03%
	MAIDE3	88.57%	95.45%	94.62%	95.61%

^a^MA: $\textbf{y}=\textbf{X}\varvec{\upbeta }+\textbf{Za}+\varvec{\upepsilon }$ ; MAI: $\textbf{y}=\textbf{X}\varvec{\upbeta }+\textbf{fb}+\textbf{Za}+\varvec{\upepsilon }$; MAE: $\textbf{y}=\textbf{X}\varvec{\upbeta }+\textbf{Za}+\textbf{Z}{\varvec{e}}_{\textbf{aa}}+\varvec{\upepsilon }$; MAIE: $\textbf{y}=\textbf{X}\varvec{\upbeta }+\textbf{fb}+\textbf{Za}+\textbf{Z}{\varvec{e}}_{\textbf{aa}}+\varvec{\upepsilon }$; MAD: $\textbf{y}=\textbf{X}\varvec{\upbeta }+\textbf{Za}+\textbf{Zd}+\varvec{\upepsilon }$; MAID: $\textbf{y}=\textbf{X}\varvec{\upbeta }+\textbf{fb}+\textbf{Za}+\textbf{Zd}+\varvec{\upepsilon }$; MADE1: $\textbf{y}=\textbf{X}\varvec{\upbeta }+\textbf{Za}+\textbf{Zd}+\textbf{Z}{\varvec{e}}_{\textbf{aa}}+\varvec{\upepsilon }$; MAIDE1: $\textbf{y}=\textbf{X}\varvec{\upbeta }+\textbf{fb}+\textbf{Za}+\textbf{Zd}+\textbf{Z}{\varvec{e}}_{\textbf{aa}}+\varvec{\upepsilon }$; MADE2: $\textbf{y}=\textbf{X}\varvec{\upbeta }+\textbf{Za}+\textbf{Zd}+\textbf{Z}{\varvec{e}}_{\textbf{aa}}+\textbf{Z}{\varvec{e}}_{\textbf{ad}}+\varvec{\upepsilon }$; MAIDE2: $\textbf{y}=\textbf{X}\varvec{\upbeta }+\textbf{fb}+\textbf{Za}+\textbf{Zd}+\textbf{Z}{\varvec{e}}_{\textbf{aa}}+\textbf{Z}{\varvec{e}}_{\textbf{ad}}+\varvec{\upepsilon }$; MADE3: $\textbf{y}=\textbf{X}\varvec{\upbeta }+\textbf{Za}+\textbf{Zd}+\textbf{Z}{\varvec{e}}_{\textbf{aa}}+\textbf{Z}{\varvec{e}}_{\textbf{ad}}+\textbf{Z}{\varvec{e}}_{\textbf{dd}}+\varvec{\upepsilon }$; MAIDE3:$\textbf{y}=\textbf{X}\varvec{\upbeta }+\textbf{fb}+\textbf{Za}+\textbf{Zd}+\textbf{Z}{\varvec{e}}_{\textbf{aa}}+\textbf{Z}{\varvec{e}}_{\textbf{ad}}+\textbf{Z}{\varvec{e}}_{\textbf{dd}}+\varvec{\upepsilon }$

^b^Model MAIDE3 did not converge for T3.

The values of Akaike Information Criterion (AIC) (Table S1) suggested that the inclusion of epistasis in the prediction models for T2, T3, and T5 improved the predictive performance of the models. Although, the Likelihood Ratio Test (LRT; Table S2) for the models fitted for the purebred pig population showed that the inclusion of epistasis was significant for the traits T2, T3 and T5, and incorporating dominance effect was also significant for T5.

### Dataset 2: crossbred pig population

#### Variance components and heritability estimates

We also estimated variance components and heritabilities for heat tolerance indicators in a crossbred (Large White x Landrace) population. Table 5 presents variance component estimates and Table 6 presents the estimates of heritability and non-additive genetic variance ratios for traits related to heat stress response in lactating sows for models including or not non-additive genetic effects. All heat stress related traits have substantial additive genetic variability (Table 5) with heritability estimates ranging from 0.0231 to 0.2639 (Table 6). However, all traits had small non-additive genetic variance with large standard error estimates, except panting score (PS) and hair density (HD) (Table 5). PS presented a ${h}_{aa}^{2}$ estimate of 0.1342, which corresponds to 82.22% of the total genetic variance for both models including epistasis (MAIEpe and MAIDEpe). HD presented ${h}_{aa}^{2}$ estimates of 0.4232 and 0.4493 for MAIE and MAIDE models, respectively. The proportion of the total genetic variance explained by additive-by-additive epistasis for HD ranged from 64.95 to 69.59% for the MAIE and MAIDE models, respectively (Table 6).

**Table 5:** Variance components for traits related to heat tolerance based on models fitting non-additive genetic effects

Trait^a^	Model^b^	$\widehat{{\sigma }_{a}^{2}}$	$\widehat{{\sigma }_{d}^{2}}$	$\widehat{{\sigma }_{aa}^{2}}$	$\widehat{{\sigma }_{pe}^{2}}$	$\widehat{{\sigma }_{e}^{2}}$
T_Vall_	MAIpe	0.0626 ± 0.0107	-	-	0.1274 ± 0.0081	0.3047 ± 0.0005
	MAIDpe	0.0618 ± 0.0111	0.0050 ± 0.0140	-	0.1237 ± 0.0128	0.3047 ± 0.0005
	MAIDEpe	0.0616 ± 0.0121	0.0047 ± 0.0144	0.0031 ± 0.0340	0.1213 ± 0.0294	0.3047 ± 0.0005
	MAIEpe	0.0618 ± 0.0120		0.0050 ± 0.0336	0.1231 ± 0.0290	0.3047 ± 0.0005
T_V4days_	MAIpe	0.0653 ± 0.0113	-	-	0.1266 ± 0.0086	0.1378 ± 0.0014
	MAIDpe	0.0642 ± 0.0116	0.0068 ± 0.0145	-	0.1216 ± 0.0132	0.1378 ± 0.0014
	MAIDEpe	0.0642 ± 0.0116	0.0068 ± 0.0145	3.48 × 10^−9^ ± 0.00000	0.1216 ± 0.0132	0.1378 ± 0.0014
	MAIEpe	0.0653 ± 0.0113	-	3.48 × 10^−9^ ± 0.00000	0.1266 ± 0.0086	0.1378 ± 0.0014
T_ES_	MAIpe	0.0316 ± 0.0069	-	-	0.0507 ± 0.0059	0.7287 ± 0.0074
	MAIDpe	0.0316 ± 0.0069	1.84 × 10^−8^ ± 0.00000	-	0.0507 ± 0.0059	0.7287 ± 0.0074
	MAIDEpe	0.0316 ± 0.0069	1.84 × 10^−8^ ± 0.00000	1.84 × 10^−8^ ± 0.00000	0.0507 ± 0.00589	0.7287 ± 0.0074
	MAIEpe	0.0316 ± 0.0069	-	1.84 × 10^−8^ ± 0.00000	0.0507 ± 0.00589	0.7287 ± 0.0074
T_SS_	MAIpe	0.0445 ± 0.0100	-	-	0.1176 ± 0.0090	0.6119 ± 0.0062
	MAIDpe	0.0437 ± 0.0104	0.0045 ± 0.0165	-	0.1145 ± 0.0145	0.6119 ± 0.0062
	MAIDEpe	0.0437 ± 0.0104	0.0045 ± 0.0165	1.54 × 10^−7^ ± 0.00000	0.1145 ± 0.0145	0.6119 ± 0.0062
	MAIEpe	0.0445 ± 0.0100	-	1.54 × 10^−7^ ± 0.00000	0.1176 ± 0.0090	0.6119 ± 0.0062
T_RS_	MAIpe	0.0276 ± 0.0063	-	-	0.0739 ± 0.0056	0.3577 ± 0.0036
	MAIDpe	0.0276 ± 0.0063	9.05 × 10^−9^ ± 0.00000	-	0.0739 ± 0.0056	0.3577 ± 0.0036
	MAIDEpe	0.0276 ± 0.0063	9.05 × 10^−9^ ± 0.00000	9.05 × 10^−9^ ± 0.00000	0.0739 ± 0.0056	0.3577 ± 0.0036
	MAIEpe	0.0276 ± 0.0063	-	9.05 × 10^−9^ ± 0.00000	0.0739 ± 0.0056	0.3577 ± 0.0036
T_TS_	MAIpe	0.0283 ± 0.0067	-	-	0.0730 ± 0.0060	0.4525 ± 0.0046
	MAIDpe	0.0278 ± 0.0070	0.0021 ± 0.0096	-	0.0716 ± 0.0089	0.4525 ± 0.0046
	MAIDEpe	0.0278 ± 0.0070	0.0021 ± 0.0096	1.14 × 10^−8^ ± 0.00000	0.0716 ± 0.00889	0.4525 ± 0.0046
	MAIEpe	0.0283 ± 0.0067	-	1.14 × 10^−8^ ± 0.00000	0.0730 ± 0.0060	0.4525 ± 0.0046
RR	MAIpe	34.7580 ± 7.0790	-	-	70.3639 ± 5.9429	442.7150 ± 4.4814
	MAIDpe	34.7580 ± 7.0790	1.11 × 10^−5^ ± 0.00000	-	70.3639 ± 5.9429	442.7150 ± 4.4814
	MAIDEpe	34.7580 ± 7.0790	1.11 × 10^−5^ ± 0.00000	1.11 × 10^−5^ ± 0.00000	70.3639 ± 5.9429	442.7150 ± 4.4814
	MAIEpe	34.7580 ± 7.0790	-	1.11 × 10^−5^ ± 0.00000	70.3639 ± 5.9429	442.7150 ± 4.4814
PS	MAIpe	0.0551 ± 0.0185	-	-	0.1297 ± 0.0195	0.8463 ± 0.0191
	MAIDpe	0.0542 ± 0.0194	0.0044 ± 0.0291	-	0.1267 ± 0.0282	0.8463 ± 0.0191
	MAIDEpe	0.0298 ± 0.0204	2.14 × 10^−8^ ± 0.00000	0.1379 ± 0.0711	0.0137 ± 0.0623	0.8463 ± 0.0191
	MAIEpe	0.0298 ± 0.0204	-	0.1379 ± 0.0711	0.0137 ± 0.0623	0.8463 ± 0.0191
HD	MAI	0.1088 ± 0.0247	-	-	-	0.3052 ± 0.0206
	MAID	0.1040 ± 0.0256	0.0289 ± 0.0366	-	-	0.2854 ± 0.0324
	MAIDE1	0.0795 ± 0.0278	0.0147 ± 0.0367	0.1745 ± 0.1015	-	0.1437 ± 0.0876
	MAIE	0.0806 ± 0.0275	-	0.1845 ± 0.0997	-	0.1455 ± 0.0876

$\widehat{{\sigma }_{a}^{2}}$: additive genetic variance estimate

$\widehat{{\sigma }_{d}^{2}}$: dominance variance estimate

$\widehat{{\sigma }_{aa}^{2}}$: additive-by-additive epistatic variance estimate

$\widehat{{\sigma }_{pe}^{2}}$: permanent environmental variance estimate

$\widehat{{\sigma }_{e}^{2}}$: residual variance estimate

^a^T_Vall_: all measures (every 10 min) of vaginal temperatures during four days (°C); T_V4days_: four-time measures of vaginal temperatures during four days (°C); T_ES_: ear skin temperature; T_SS_: shoulder skin temperature; T_RS_: rump skin temperature; T_TS_: tail skin temperature; RR: respiration rate; PS: panting score; HD: hair density.

^b^MAIpe: $\textbf{y}=\textbf{X}\varvec{\upbeta }+\textbf{fb}+\textbf{Za}+\textbf{Zpe}+\varvec{\upepsilon }$; MAIEpe: $\textbf{y}=\textbf{X}\varvec{\upbeta }+\textbf{fb}+\textbf{Za}+\textbf{Zpe}+\textbf{Z}{\varvec{e}}_{\textbf{aa}}+\varvec{\upepsilon }$; MAIDpe: $\textbf{y}=\textbf{X}\varvec{\upbeta }+\textbf{fb}+\textbf{Za}+\textbf{Zpe}+\textbf{Zd}+\varvec{\upepsilon }$; MAIDEpe: $\textbf{y}=\textbf{X}\varvec{\upbeta }+\textbf{fb}+\textbf{Za}+\textbf{Zd}+\textbf{Z}{\varvec{e}}_{\textbf{aa}}+\textbf{Zpe}+\varvec{\upepsilon }$; MAI: $\textbf{y}=\textbf{X}\varvec{\upbeta }+\textbf{fb}+\textbf{Za}+\varvec{\upepsilon }$; MAIE: $\textbf{y}=\textbf{X}\varvec{\upbeta }+\textbf{fb}+\textbf{Za}+\textbf{Z}{\varvec{e}}_{\textbf{aa}}+\varvec{\upepsilon }$; MAID: $\textbf{y}=\textbf{X}\varvec{\upbeta }+\textbf{fb}+\textbf{Za}+\textbf{Zd}+\varvec{\upepsilon }$; MAIDE1:$\textbf{y}=\textbf{X}\varvec{\upbeta }+\textbf{fb}+\textbf{Za}+\textbf{Zd}+\textbf{Z}{\varvec{e}}_{\textbf{aa}}+\varvec{\upepsilon }$

**Table 6:** Heritability estimates and variance ratios considering models fitting non-additive genetic effects for heat tolerance indicators

Trait^a^	Model^b^	${h}_{a}^{2}$	${h}_{d}^{2}$	${h}_{aa}^{2}$	$\widehat{{\sigma }_{d}^{2}}/\widehat{{\sigma }_{g}^{2}}$	$\widehat{{\sigma }_{aa}^{2}}/\widehat{{\sigma }_{g}^{2}}$
T_Vall_	MAIpe	0.1266 ± 0.0203	-	-	-	-
	MAIDpe	0.1248 ± 0.0211	0.0102 ± 0.0285	-	0.0753 ± 0.1990	-
	MAIDEpe	0.1239 ± 0.0232	0.0096 ± 0.0290	0.0062 ± 0.0670	0.0686 ± 0.2038	0.0442 ± 0.4633
	MAIEpe	0.1249 ± 0.0229	-	0.0102 ± 0.0660	-	0.0754 ± 0.4577
T_V4days_	MAIpe	0.1982 ± 0.0310	-	-	-	-
	MAIDpe	0.1944 ± 0.0323	0.0206 ± 0.0441	-	0.0959 ± 0.1898	-
	MAIDEpe	0.1944 ± 0.0323	0.0206 ± 0.0441	0.0000 ± 0.0000	0.0959 ± 0.1898	0.0000 ± 0.0000
	MAIEpe	0.1982 ± 0.0310	-	0.0000 ± 0.0000	-	0.0000 ± 0.0000
T_ES_	MAIpe	0.039 ± 0.0083	-	-	-	-
	MAIDpe	0.039 ± 0.0083	0.0000 ± 0.0000		0.0000 ± 0.0000	-
	MAIDEpe	0.039 ± 0.0083	0.0000 ± 0.0000	0.0000 ± 0.0000	0.0000 ± 0.0000	0.0000 ± 0.0000
	MAIEpe	0.039 ± 0.0083	-	0.0000 ± 0.0000	-	0.0000 ± 0.0000
T_SS_	MAIpe	0.0575 ± 0.0126	-	-	-	-
	MAIDpe	0.0564 ± 0.0132	0.0058 ± 0.0211	-	0.0925 ± 0.3148	-
	MAIDEpe	0.0564 ± 0.0132	0.0058 ± 0.0211	0.0000 ± 0.0000	0.0925 ± 0.3148	0.0000 ± 0.0000
	MAIEpe	0.0575 ± 0.0126	-	0.0000 ± 0.0000	-	0.0000 ± 0.0000
T_RS_	MAIpe	0.0601 ± 0.0134	-	-	-	-
	MAIDpe	0.0601 ± 0.0134	0.0000 ± 0.0000	-	0.0000 ± 0.0000	
	MAIDEpe	0.0601 ± 0.0134	0.0000 ± 0.0000	0.0000 ± 0.0000	0.0000 ± 0.0000	0.0000 ± 0.0000
	MAIEpe	0.0601 ± 0.0134	-	0.0000 ± 0.0000		0.0000 ± 0.0000
T_TS_	MAIpe	0.051 ± 0.0119	-	-	-	-
	MAIDpe	0.0501 ± 0.0124	0.0038 ± 0.0174	-	0.0708 ± 0.3052	-
	MAIDEpe	0.0501 ± 0.0124	0.0038 ± 0.0174	0.0000 ± 0.0000	0.0708 ± 0.3052	0.0000 ± 0.0000
	MAIEpe	0.0501 ± 0.0119	-	0.0000 ± 0.0000	-	0.0000 ± 0.0000
RR	MAIpe	0.0634 ± 0.0126	-	-	-	-
	MAIDpe	0.0634 ± 0.0126	0.0000 ± 0.0000	-	0.0000 ± 0.0000	-
	MAIDEpe	0.0634 ± 0.0126	0.0000 ± 0.0000	0.0000 ± 0.0000	0.0000 ± 0.0000	0.0000 ± 0.0000
	MAIEpe	0.0634 ± 0.0126	-	0.0000 ± 0.0000		0.0000 ± 0.0000
PS	MAIpe	0.0535 ± 0.0176	-	-		-
	MAIDpe	0.0526 ± 0.0185	0.0042 ± 0.0286	-	0.0744 ± 0.4744	-
	MAIDEpe	0.0290 ± 0.0198	0.0000 ± 0.0000	0.1342 ± 0.0689	0.0000 ± 0.0000	0.8222 ± 0.1527
	MAIEpe	0.0290 ± 0.0198	-	0.1342 ± 0.0689	-	0.8222 ± 0.1527
HD	MAI	0.2629 ± 0.0535	-	-	-	-
	MAID	0.2494 ± 0.0567	0.0662 ± 0.0869	-	0.2097 ± 0.2335	-
	MAIDE1	0.1928 ± 0.0641	0.0356 ± 0.0875	0.4232 ± 0.2468	0.0546 ± 0.1338	0.6495 ± 0.2020
	MAIE	0.1964 ± 0.0632	-	0.4493 ± 0.2419	-	0.6959 ± 0.1617

${h}_{a}^{2}$: additive heritability or narrow-sense heritability

${h}_{d}^{2}$: dominance variance ratio

${h}_{aa}^{2}$: epistatic additive-by-additive variance ratio

$\widehat{{\sigma }_{d}^{2}}/\widehat{{\sigma }_{g}^{2}}$: ratio of the total genetic variance explained by dominance

$\widehat{{\sigma }_{aa}^{2}}/\widehat{{\sigma }_{g}^{2}}$: ratio of the total genetic variance explained by additive-by-additive epistasis

^a^T_Vall_: all measures (every 10 min) of vaginal temperatures during four days (°C); T_V4days_: four-time measures of vaginal temperatures during four days (°C); T_ES_: ear skin temperature; T_SS_: shoulder skin temperature; T_RS_: rump skin temperature; T_TS_: tail skin temperature; RR: respiration rate; PS: panting score; HD: hair density.

^b^ MAIpe: $\textbf{y}=\textbf{X}\varvec{\upbeta }+\textbf{fb}+\textbf{Za}+\textbf{Zpe}+\varvec{\upepsilon }$; MAIEpe: $\textbf{y}=\textbf{X}\varvec{\upbeta }+\textbf{fb}+\textbf{Za}+\textbf{Zpe}+\textbf{Z}{\varvec{e}}_{\textbf{aa}}+\varvec{\upepsilon }$; MAIDpe: $\textbf{y}=\textbf{X}\varvec{\upbeta }+\textbf{fb}+\textbf{Za}+\textbf{Zpe}+\textbf{Zd}+\varvec{\upepsilon }$; MAIDEpe: $\textbf{y}=\textbf{X}\varvec{\upbeta }+\textbf{fb}+\textbf{Za}+\textbf{Zd}+\textbf{Z}{\varvec{e}}_{\textbf{aa}}+\textbf{Zpe}+\varvec{\upepsilon }$; MAI: $\textbf{y}=\textbf{X}\varvec{\upbeta }+\textbf{fb}+\textbf{Za}+\varvec{\upepsilon }$; MAIE: $\textbf{y}=\textbf{X}\varvec{\upbeta }+\textbf{fb}+\textbf{Za}+\textbf{Z}{\varvec{e}}_{\textbf{aa}}+\varvec{\upepsilon }$; MAID: $\textbf{y}=\textbf{X}\varvec{\upbeta }+\textbf{fb}+\textbf{Za}+\textbf{Zd}+\varvec{\upepsilon }$; MAIDE1:$\textbf{y}=\textbf{X}\varvec{\upbeta }+\textbf{fb}+\textbf{Za}+\textbf{Zd}+\textbf{Z}{\varvec{e}}_{\textbf{aa}}+\varvec{\upepsilon }$

As observed for the purebred population (Dataset 1), the inclusion of non-additive genetic effects in the model did not affect the residual variance estimates for most of the traits, except for HD (Table 5). However, for HD and PS there was also a decrease in the additive genetic variance estimates (Table 5) when epistasis was included in the model. When both dominance and epistasis were included in the repeatability model (for all heat stress-related traits, except HD), there was a decrease in the permanent environmental variance estimate for most of the traits.

### Model comparison for the crossbred pig dataset

For the crossbred population, the models also presented similar values of Akaike Information Criterion (AIC) (Table S3). LRT (Table S4) for the models fitted for the crossbred pig population showed that fitting epistasis in the models had a significant effect only for PS. The models for the other traits did not have better goodness of fit when dominance and/or epistasis were fitted.

## Discussion

We aimed to estimate variance components and evaluate the predictive ability of genomic models including non-additive genetic effects for different traits in purebred and crossbred populations. In general, we observed small estimates for dominance variance and the inclusion of additive-by-additive epistasis in the model reduced the estimates of additive genetic variance. The inclusion of non-additive genetic effects in models for genomic prediction resulted in changes in animals’ ranking and in the groups of animals to be selected. Since two different independent populations were used in this study, the Discussion section was structured into two subsections to discuss the findings for each population separately.

### Dataset 1: purebred pig population

One way to account for inbreeding depression in genomic prediction is by fitting the inbreeding coefficient as a covariate in the model. The benefit of doing so is that unbiased variance components may be obtained because the variance component estimates may be inflated when inbreeding or heterozygosity are not fitted within the models [17–19]. According to Bolormaa et al. [4] and Xiang et al. [19], inbreeding or heterozygosity are fitted within the models to account for directional dominance (i.e., a higher percentage of positive than negative dominance effects), otherwise, the variance components for the dominance effects will be biased. Vitezica et al. [17] also reported that in the absence of inbreeding within the models, the estimates for dominance variance were inflated for litter size in a purebred pig line. In the present study, there was a slight decrease in the additive genetic variance when the models adjusted for inbreeding depression, suggesting that not fitting inbreeding in the model might generate overestimated additive genetic variances.

The heritability estimates obtained from the models including only the additive and dominance genetic effects (MAD) corroborate with the estimates reported in previous studies using the same public dataset [32]. The studies reported dominance variance aiming to propose different methods for obtaining additive and dominance genetic variance components [33–35]. The dominance variance ratio estimates for T3 reported by Da et al. [33] and Liu et al. [35] were greater than estimated in this study (0.07 vs. 0.02), and the dominance variance ratio estimated for T5 was smaller than that one reported by Nishio and Satoh [34] (0.045 vs. 0.063). Da et al. [36] proposed multifactorial methods with SNP and haplotypes to calculate the genomic relationship matrix and fitted the epistasis effects up to third-order in the genomic prediction models. When comparing the estimates from the most complete models, in general, Da et al. [36] reported similar heritability (0.02 vs. 0.01, 0.22 vs. 0.21, 0.13 vs. 0.12, 0.33 vs. 0.31, 0.36 vs. 0.34 for T1, T2, T3, T4, and T5, respectively) and lower additive-by-additive epistatic variance ratio estimates (0.04 vs. 0.13, 0.18 vs. 0.24, 0.28 vs. 0.29, 0.02 vs. 0.09, 0.05 vs. 0.13 for T1, T2, T3, T4, and T5, respectively). The differences between estimates reported by each study using the same dataset might be due to different methods and/or parametrization considered to obtain the genomic relationship matrices in each study.

The method to obtain the relationship matrices applied in this study, proposed by Vitezica et al. [6], is a flexible approach that can also be applied for populations that are not in Hardy-Weinberg equilibrium. However, it assumes linkage equilibrium between the genetic markers to ensure orthogonality between variance components. The non-orthogonality is clearly observed when there are significant changes in the variance estimates and when new variance components are introduced into the model [6]. In this study, the non-orthogonality between the variance components was noticeable when epistasis, especially additive-by-additive, was added to the model. This source of non-orthogonality might be due to linkage disequilibrium (LD) between the markers since this first dataset is from a nucleus purebred pig line, which is under intense selection pressure for various traits. Vitezica et al. [6] also reported high additive-by-additive variance and a sum of non-additive variances greater than the additive variance for a simulated high LD scenario due to strong selection. The additive, dominance, and epistasis effects are assumed independent of each other under the linkage equilibrium assumption [37, 38]. However, the presence of LD might affect the magnitude of dominance and epistasis variance due to the correlation between markers by introducing dependency between the genetic additive and non-additive values, which could lead to overestimation of the non-additive genetic variance components. As a result, the partition between additive and non-additive components is more difficult when LD between markers is present [39]. It is worth noting that many complex traits are predominantly additive [40], and the additive genetic variance often comprises most of the total genetic variance for most traits [41]. Accurate estimation of additive genetic variance components can be achieved without assuming linkage equilibrium. Therefore, incorporating non-additive genetic effects into genomic prediction may not offer significant advantages for selection purposes. However, to evaluate the genetic architecture of complex traits, there is still a need to develop methods for accurately estimating non-additive genetic variance components without assuming linkage equilibrium, especially considering that many livestock populations are subjected to intensive selective breeding (a major cause of LD).

As summarized by Wade et al. [42], for two bi-allelic loci, epistasis can be considered in different ways: (i) additive-by-additive, interactions between homozygotes at both loci; (ii) additive-by-dominance, interactions between homozygotes at locus A and heterozygotes at locus B and vice-versa; (iii) dominance-by-dominance, interactions between heterozygotes at both loci. The first dataset used in this study belongs to a purebred population, which could be one of the reasons why the variance components for additive-by-dominance and dominance-by-dominance epistasis are close to zero. Purebred populations tend to exhibit reduced heterozygosity, which can make it more challenging to accurately estimate additive-by-dominance and dominance-by-dominance epistatic effects [43]. The results indicate that although there may be those types of interactions between loci in the purebred population evaluated, they might be mostly captured by the additive genetic variance [41].

All models presented dispersion close to one, indicating that there is no inflation or deflation for the genomic estimated breeding values (GEBVs) [30]. The GEBVs also presented bias close to zero in all models, which is desirable, since biased estimates compromise the estimates of genetic trends and genetic gains in breeding programs [44]. However, the accuracy differed across traits and traits with higher (additive) heritability yielded greater accuracies, which agrees with the literature [45–47]. Different accuracies were estimated for each model, and there was a decrease in the accuracy when epistasis effects were incorporated in the models, which can be associated with the decrease in the additive genetic variance components for those models, since this variance component are used to compute the accuracy [44].

Although models including epistatic effects were significantly better (based on LRT parameter) for some traits (T2, T3, and T5), including epistasis had lower GEBV accuracies for traits with lower heritability (T1, T2, and T3). It is noticeable that models including epistatic effects had greater standard errors for the additive genetic variance and heritability estimates for T1, which is the trait with the lowest heritability. This suggests that the available data might not be sufficient to effectively distinguish between additive genetic and epistasis effects. Therefore, larger datasets may be required to obtain more accurate estimates of non-additive genetic effects [22], particularly for traits with low heritability.

Another factor influencing this inferior accuracy for models including epistasis may be due to the non-orthogonality between the components, especially between additive genetic effects and additive-by-additive epistasis. At the same time, when only additive genetic effects were fitted in the models, part of the additive-by-additive epistasis was captured by the additive genetic component, as previously reported in other studies [3, 48]. Even though models including additive-by-additive epistasis was significantly better (based on LRT parameter) and this effect accounted for a relevant proportion of phenotypic variance for some traits in the purebred population, including epistasis in the model may capture part of the additive genetic variance [3]. The ranking of selection candidates also changed for models as a consequence of the change in variance partition due to the inclusion of non-additive genetic effects in the models.

In general, models including epistasis presented the largest changes in ranking of the animals in comparison to the additive model. According to Piccoli et al. [49], the degree of reranking is directly associated with the accuracies of estimates and, at the same time, it is difficult to compare the reranking considering real datasets since the true breeding values are unknown. However, it is noticeable that epistasis plays an important role in genomic prediction and might change the selected animals when considered in a genetic evaluation program, which would impact genetic progress for the traits evaluated. There are studies investigating the impact of incorporating epistasis into genomic predictions aiming to develop methods that effectively integrate this effect into genomic prediction models (e.g., [50, 51]). Previous studies have shown that including all possible marker interactions in the prediction model does not improve the prediction ability [52, 53]. However, there is an improvement in prediction ability when only some interactions between markers are included in the model, being selected only those interactions with greater effects [52, 53].

### Dataset 2: crossbred pig population

Since all heat stress-related traits had substantial additive genetic variability, including them in a selection program is feasible and can lead to genetic progress. Although, it should be noted that phenotypes measured in crossbred animals are expected to be more influenced by non-additive genetic effects than in purebred populations [21, 22]. Small non-additive genetic variances were observed for most of the traits evaluated in the crossbred dataset. Additionally, the estimates for dominance and epistasis variances had large standard errors which illustrates the difficulty of obtaining good estimates [17] and suggesting that the dataset was not large enough [19].

Besides HD and PS having considerable epistasis estimates, models including epistatic effects were significant better (based on LRT parameter) only for PS and no significant dominance variance was observed for any trait related to heat stress in the crossbred population. However, HD and PS are categorical traits and, threshold models [54–56] should be more suitable for genetically evaluating them. As highlighted by Alves et al. [3], there might be some limitations in estimating non-additive genetic effects for categorical traits.

Estimating non-additive genetic variances is also difficult, since they are often, or at least partially, confounded with other effects, such as common environmental or maternal effects [4]. In this study, it is noticeable that, for traits related to heat stress, there was a confoundment between permanent environmental and non-additive genetic effects. This was observed through a reduction in the variance of permanent environmental effects when non-additive genetic effects were included in the model. This observation, particularly regarding PS, is similar to the results reported by Vitezica et al. [17]. The authors also reported that including non-additive genetic effects in the model did not have a large impact on residual variances and the non-additive genetic effects were captured by the permanent environmental effects [17].

The findings from this study suggest that the gene actions for heat stress-related traits in this population are mainly additive, or at least most of the non-additive genetic effects are captured by the additive genetic component. Although the dataset used in this study came only from crossbred animals, the pure breeds used for crossbreeding are both considered maternal breeds (Large White and Landrace) and genetically related [57–59]. Thus, in crossings between lines or breeds with high genetic distance it would be expected higher heterosis and greater non-additive genetic variance estimates [21] in comparison to the present study.

### Implications and future studies

Quantitative traits are controlled by many genes with additive and potentially non-additive gene actions, and their phenotypic expression are the result of many developmental and biochemical pathways comprised of loci networks that interact at the genetic and molecular levels, generating the epistasis effects [60]. Thus, the magnitude of the epistasis effects depends on how many pathways are involved in the expression of certain phenotypes and how these pathways are connected and interact between them. In the other hand, the magnitude of dominance effects depends on the deviations of genotypic values from breeding values for each locus. If the proportion of positive dominance effects is greater than the proportion of negative dominance effects, it is referred to as directional dominance [1, 9]. Heterosis and inbreeding depression are directly dependent on non-additive genetic effects [9, 17]. Although it is expected that non-additive genetic variation would be larger in crossbred populations [5, 9, 22, 61], most of the traits measured in the crossbred population did not present substantial non-additive genetic variance ratios. Additionally, fitting non-additive genetic effects in the models did not improve the prediction ability of breeding values for the purebred population. However, including non-additive genetic effects, especially additive-by-additive epistasis, changed the animal’s ranking and can therefore affect selection decisions.

Many studies have suggested that non-additive genetic effects are greater in traits related to fitness and adaptation than in morphological traits [62–65]. Considering the importance of selecting more climatically resilient animals, it is necessary to elucidate the genetic background of traits related to heat stress, especially for crossbred pigs. In our study, although models including non-additive genetic effects were not significant (based on LRT parameter) for most of the traits related to heat stress response, these effects might play an important role in gene action for those traits. Thus, it would be important to perform studies considering a larger dataset including records from purebred and crossbred populations and evaluate different crossings as well.

## Conclusions

Including dominance effects in genomic prediction models did not improve the predictive ability of the models and most traits had none to low dominance variance. In the purebred population, low dominance variance ratios were observed, potentially due to reduced heterozygosity. Although models including additive-by-additive epistasis effects in genomic prediction exhibited significantly better fit for some traits, it led to lower accuracy when genomically predicting breeding values for traits with low heritability. For the evaluated populations and models, the inclusion of non-additive genetic effects did not improve genomic prediction accuracy. The non-orthogonality between variance components, especially between additive and additive-by-additive epistasis, suggests the presence of linkage disequilibrium challenges in the partition of additive and non-additive genetic effects. Therefore, there is still a need for developing methods able to accurately estimate non-additive variance components without assuming linkage equilibrium.

Traits related to heat stress in the crossbred population had substantial additive genetic variability, indicating their suitability for inclusion in selection programs. The difficulty in estimating non-additive genetic effects and the confounding with other effects, such as permanent environmental effects, is a challenge to obtain accurate estimates. Models incorporating non-additive genetic effects in genomic prediction for heat stress-related traits did not perform better than the additive model for most of the traits evaluated. Future studies should use larger datasets when they become available, including both purebred and crossbred animals, to better understand the contribution of non-additive genetic effects, particularly for traits related to adaptation, welfare, and resilience.

## Methods

### Dataset 1: purebred pig population

#### Purebred dataset description

We used a public dataset [32] including pedigree, genotypic, and phenotypic information from a single nucleus pig line from Pig Improvement Company (PIC, Hendersonville, TN, USA). This dataset consisted of 3,534 individuals with genotypes for 52,843 SNPs and phenotypes for five performance traits that represent a small number of phenotypes that are routinely collected from birth in the genetic nucleus and with heritability estimates ranging from 0.07 to 0.62. The phenotype was either pre-corrected for environmental factors and rescaled by correcting for the overall mean (traits 3, 4, and 5) or was a rescaled, weighted mean of corrected progeny phenotypes (traits 1 and 2). The data also includes a pedigree file with parents and grandparents of the genotyped animals [32]. The descriptive statistics of the traits are presented in Table 7.

**Table 7:** Descriptive statistics of a public dataset from a purebred pig population [32]

Trait	Number of observations	Mean	SD^a^
T1	2,804	-0.0452	1.2077
T2	2,715	0.0049	1.1230
T3	3,141	0.7058	0.9607
T4	3,152	-1.0726	2.3276
T5	3,184	37.9888	60.4468

^a^SD: standard deviation

The quality control of genotype data consisted of removing SNPs with call rate lower than 0.90, minor allele frequency (MAF) less than 0.01, and extreme deviation from Hardy-Weinberg equilibrium (p-value < 10^−5^). After quality control, 40,828 SNPs and 3,534 individuals remained for further analyses. No animals were removed during the quality control due their genotypes were imputed [32], then all animals presented higher call rate (> 0.90).

#### Variance components estimation

Different models including or not inbreeding and non-additive genetic effect of dominance and epistasis were fitted to estimate variance components:

- MA: $$\textbf{y}=\textbf{X}\varvec{\upbeta }+\textbf{Za}+\varvec{\upepsilon }$$;
- MAI: $$\textbf{y}=\textbf{X}\varvec{\upbeta }+\textbf{fb}+\textbf{Za}+\varvec{\upepsilon }$$;
- MAE: $$\textbf{y}=\textbf{X}\varvec{\upbeta }+\textbf{Za}+\textbf{Z}{\textbf{e}}_{\textbf{aa}}+\varvec{\upepsilon }$$;
- MAIE: $$\textbf{y}=\textbf{X}\varvec{\upbeta }+\textbf{fb}+\textbf{Za}+\textbf{Z}{\textbf{e}}_{\textbf{aa}}+\varvec{\upepsilon }$$;
- MAD: $$\textbf{y}=\textbf{X}\varvec{\upbeta }+\textbf{Za}+\textbf{Zd}+\varvec{\upepsilon }$$;
- MAID: $$\textbf{y}=\textbf{X}\varvec{\upbeta }+\textbf{fb}+\textbf{Za}+\textbf{Zd}+\varvec{\upepsilon }$$;
- MADE1: $$\textbf{y}=\textbf{X}\varvec{\upbeta }+\textbf{Za}+\textbf{Zd}+\textbf{Z}{\textbf{e}}_{\textbf{aa}}+\varvec{\upepsilon }$$;
- MAIDE1: $$\textbf{y}=\textbf{X}\varvec{\upbeta }+\textbf{fb}+\textbf{Za}+\textbf{Zd}+\textbf{Z}{\textbf{e}}_{\textbf{aa}}+\varvec{\upepsilon }$$;
- MADE2: $$\textbf{y}=\textbf{X}\varvec{\upbeta }+\textbf{Za}+\textbf{Zd}+\textbf{Z}{\textbf{e}}_{\textbf{aa}}+\textbf{Z}{\textbf{e}}_{\textbf{ad}}+\varvec{\upepsilon }$$;
- MAIDE2: $$\textbf{y}=\textbf{X}\varvec{\upbeta }+\textbf{fb}+\textbf{Za}+\textbf{Zd}+\textbf{Z}{\textbf{e}}_{\textbf{aa}}+\textbf{Z}{\textbf{e}}_{\textbf{ad}}+\varvec{\upepsilon }$$;
- MADE3: $$\textbf{y}=\textbf{X}\varvec{\upbeta }+\textbf{Za}+\textbf{Zd}+\textbf{Z}{\textbf{e}}_{\textbf{aa}}+\textbf{Z}{\textbf{e}}_{\textbf{ad}}+\textbf{Z}{\textbf{e}}_{\textbf{dd}}+\varvec{\upepsilon }$$;
- MAIDE3: $$\textbf{y}=\textbf{X}\varvec{\upbeta }+\textbf{fb}+\textbf{Za}+\textbf{Zd}+\textbf{Z}{\textbf{e}}_{\textbf{aa}}+\textbf{Z}{\textbf{e}}_{\textbf{ad}}+\textbf{Z}{\textbf{e}}_{\textbf{dd}}+\varvec{\upepsilon }$$;

where $\varvec{y}$ is the vector of observations; $\varvec{\upbeta }$ is the vector of fixed effects; $\textbf{f}$ is the vector of inbreeding coefficient based on pedigree; $\textbf{b}$ is the inbreeding depression; **a** is the vector of additive genetic effects with $\textbf{a} \sim N(0, {\textbf{G}}_{\textbf{A}}{\sigma }_{a}^{2})$, where ${\sigma }_{a}^{2}$ is the additive genetic variance and ${\textbf{G}}_{\textbf{A}}$ is the genomic additive relationship matrix; $\textbf{d}$ is the vector of dominance effects with $\textbf{d} \sim N(0, {\textbf{G}}_{\textbf{D}}{\sigma }_{d}^{2})$ where ${\sigma }_{d}^{2}$ is the dominance variance and ${\textbf{G}}_{\textbf{D}}$ the genomic dominance relationship matrix; ${\textbf{e}}_{\textbf{aa}}$ is the vector of additive-by-additive epistatic effects, ${\textbf{e}}_{\textbf{aa}} \sim N(0, {\textbf{G}}_{\textbf{AA}}{\sigma }_{aa}^{2})$, where ${\sigma }_{aa}^{2}$ is the additive-by-additive epistatic variance and ${\textbf{G}}_{\textbf{AA}}$ the genomic additive-by-additive epistatic relationship matrix; ${\textbf{e}}_{\textbf{ad}}$ is the vector of additive-by-dominance epistatic effects, ${\textbf{e}}_{\textbf{ad}} \sim N(0, {\textbf{G}}_{\textbf{AD}}{\sigma }_{ad}^{2})$, where ${\sigma }_{ad}^{2}$ is the additive-by-dominance epistatic variance and ${\textbf{G}}_{\textbf{AD}}$ the genomic additive-by-dominance epistatic relationship matrix; ${\textbf{e}}_{\textbf{dd}}$ is the vector of dominance-by-dominance epistatic effects, ${\textbf{e}}_{\textbf{dd}} \sim N(0, {\textbf{G}}_{\textbf{DD}}{\sigma }_{dd}^{2})$, where ${\sigma }_{dd}^{2}$ is the dominance-by-dominance epistatic variance and ${\textbf{G}}_{\textbf{DD}}$ the genomic dominance-by-dominance relationship matrix; $\varvec{\upepsilon }$ is the vector of residuals, $\varvec{\upepsilon } \sim N(0, \textbf{I}{\sigma }_{\epsilon }^{2})$, where ${\sigma }_{\epsilon }^{2}$ is the residual variance and $\textbf{I}$ an identity matrix; $\textbf{X}$ and $\textbf{Z}$ are incidence matrices of fixed and genetic effects, respectively.

The genomic additive relationship matrix (${\textbf{G}}_{\textbf{A}}$) was computed according to the method proposed by VanRaden [66]: ${\textbf{G}}_{\textbf{A}}=\frac{\textbf{MM}^{\prime}}{2\sum {\text{p}}_{\rm{i}}{\text{q}}_{\rm{i}}}$, where the **M** matrix contains elements equal to ($2-2{p}_{i}$), ($1-2{p}_{i}$), ($-2{p}_{i}$) for ${A}_{1}{A}_{1}$, ${A}_{1}{A}_{2,}$ and ${A}_{2}{A}_{2}$ genotypes, respectively, being ${p}_{i}$ the frequency of the allele ${A}_{1}$ in the SNP *i* and ${q}_{i}$ is equal to $1-{p}_{i}$. The genomic dominance relationship matrix (${\textbf{G}}_{\textbf{D}}$) was computed according to the method proposed by Vitezica et al. [5]: ${\textbf{G}}_{\textbf{D}}=\frac{\textbf{WW}'}{4\sum {p}_{i}^{2}{q}_{i}^{2}}$, where the **W** matrix contains elements equal to (-$2{q}_{i}^{2}$), ($2{p}_{i}{q}_{i}$), ($-2{p}_{i}^{2}$) for ${A}_{1}{A}_{1}$, ${A}_{1}{A}_{2}$, and ${A}_{2}{A}_{2}$ genotypes, respectively. The genomic epistatic relationship matrices were computed according to the methods proposed by Vitezica et al. [6]: ${\textbf{G}}_{\textbf{AA}}=\frac{{\text{G}}_{\text{A}}\odot\,{\text{G}}_{\text{A}}}{\text{tr}({\text{G}}_{\text{A}}\odot\,{\text{G}}_{\text{A}})/\text{n}}$, ${\textbf{G}}_{\textbf{AD}}=\frac{{\text{G}}_{\text{A}}\odot\,{\text{G}}_{\text{D}}}{\text{tr}({\text{G}}_{\text{A}}\odot\,{\text{G}}_{\text{D}})/\text{n}}$, and ${\textbf{G}}_{\textbf{DD}}=\frac{{\text{G}}_{\text{D}}\odot\,{\text{G}}_{\text{D}}}{\text{t}\text{r}({\text{G}}_{\text{D}}\odot\,{\text{G}}_{\text{D}})/\text{n}}$ being $\odot$ the Hadamard product, $tr$ the matrix trace, and $n$ the number of animals.

The analyses were performed using the ASREML software [67]. To understand how the phenotypic variance was split when different non-additive genetic effects were included in the models, variance components were estimated for each model. Estimates of additive heritability or narrow-sense heritability (${h}_{a}^{2}$), dominance variance ratio (${h}_{d}^{2}$), epistatic additive-by-additive variance ratio (${h}_{aa}^{2}$), epistatic additive-by-dominance variance ratio (${h}_{ad}^{2}$), and epistatic dominance-by-dominance variance ratio (${h}_{dd}^{2}$) were computed as the ratio of estimates of additive genetic variance ($\widehat{{\sigma }_{a}^{2}}$), dominance variance ($\widehat{{\sigma }_{d}^{2}}$), additive-by-additive epistatic variance ($\widehat{{\sigma }_{aa}^{2}}$), additive-by-dominance epistatic variance ($\widehat{{\sigma }_{ad}^{2}}$), and dominance-by-dominance epistatic variance ($\widehat{{\sigma }_{dd}^{2}}$) to the total phenotypic variance ($\widehat{{\sigma }_{p}^{2}}$).

##### Predictive ability

Bias, dispersion, and accuracy estimates were obtained using the Linear Regression (LR) method [44]. LR method is based on the comparison of breeding values estimated from partial datasets with a dataset containing all information (whole dataset). The whole dataset was composed of all animals and their observations, while ten partial datasets were generated by setting 10% of records missing to perform a 10-fold cross-validation. The prediction bias was computed as:

$${\mu }_{wp} = \overset{-}{{\widehat{\boldsymbol{u}}}_{p}} - \overset{-}{{\widehat{\boldsymbol{u}}}_{w}},$$

where ${\mu }_{wp}$ is the bias, $\overset{-}{{\widehat{\varvec{u}}}_{p}}$ is the GEBV mean predicted with partial information (GEBV_p_) and $\overset{-}{{\widehat{\varvec{u}}}_{w}}$ is the GEBV mean predicted with whole information (GEBV_w_). The GEBV dispersion is the slope of the regression (${b}_{w,p}$) of GEBV_w_ (${\widehat{\varvec{u}}}_{w}$) on GEBV_p_ (${\widehat{\varvec{u}}}_{p}$), and was obtained as:

$${b}_{w,p}=\frac{cov({\widehat{\varvec{u}}}_{p},{\widehat{\varvec{u}}}_{w})}{var\left({\widehat{\varvec{u}}}_{p}\right)}$$

The accuracy was obtained as:

$${acc}_{p}=\sqrt{\frac{cov({\widehat{\varvec{u}}}_{p},{\widehat{\varvec{u}}}_{w})}{\left(1-\overset{-}{F}\right)\widehat{{\sigma }_{a}^{2}}}}$$

where ${acc}_{p}$ is the accuracy, $\overset{-}{F}$ is the mean inbreeding coefficient based on pedigree from the animals with partial information, and $\widehat{{\sigma }_{a}^{2}}$ is the estimated additive genetic variance using the whole population.

###### Model comparison

The AIC [68] LRT parameters were used to compare the models. LRT was computed separately for the models that included or not the inbreeding coefficient as a covariate. This was done to specifically assess the impact of incorporating random non-additive genetic effects on the model, while isolating the influence of the inbreeding coefficient. The LRT statistic was calculated as:

LRT = − 2(LogL _reduced model_ − LogL _complete model_),

in which LogL_reduced model_ and LogL_complete model_ are the likelihood logarithms for the reduced (i.e., from additive model; MA or MAI) and complete models (i.e., from the models including non-additive genetic effects), respectively. The level of significance used was 0.05.

We also evaluated the impact of the model used on the ranking of the animals and the proportion of commonly selected individuals based on the GEBVs from different models. The Spearman’s rank correlation was calculated to evaluate the re-ranking between the GEBVs from the MA model, which is the most used approach for GEBV calculation, and the other evaluated models. Assuming different selection intensities, the top 1%, 5%, 10%, and 20% of the animals were ranked according to their GEBVs based on the MA model and compared with the animals selected based on the models including non-additive effects.

### Dataset 2: crossbred pig population

#### Crossbred dataset description

To evaluate the contribution of non-additive genetic effects on traits related to heat stress in lactating sows, we used a dataset previously described by Johnson et al. [68]. In summary, a total of 1,645 multiparous lactating sows (Large White × Landrace) were housed in either a naturally ventilated or mechanically ventilated farrowing barn. Thermoregulatory and anatomical traits were measured in 1,381 sows. The traits included in this study were skin surface temperature from ear (T_ES_), shoulder (T_SS_), rump (T_RS_), and tail (T_TS_); vaginal temperature considering the whole data measured every 10 min (T_Vall_), and the average of the six records per hour corresponding to 08:00, 12:00, 16:00, and 20:00 h during four days (T_V4days_); respiration rate (RR), panting score (PS; score scale from 0 to 3), and hair density (HD, score scale from 0 to 2) (the data collection is described in details by Johnson et al. [69]). All animals were genotyped using the PorcineSNP50K (50,703 SNPs) Bead Chip (Illumina, San Diego, CA, USA). Quality control of genotype data consisted of removing SNPs with a call rate lower than 0.90 and minor allele frequency (MAF) less than 0.01. It was also removed animals with call rate lower than 0.90. After quality control, 49,536 SNPs for 1,625 animals remained for further analyses. The descriptive statistics for the continuous traits are shown in Table 8.

**Table 8:** Descriptive statistics for continuous heat stress indicators in Landrace x Large White crossbred pig population

Trait^a^	Number of animals	Number of observations	Mean	Minimum	Maximum	Standard deviation
T_Vall_	1,381	932,708	39.74	37.08	42.35	0.76
T_V4days_	1,381	21,415	39.70	37.10	42.70	0.77
T_ES_	1,381	21,411	36.70	32.50	40.70	1.07
T_SS_	1,381	21,414	36.50	32.30	39.80	1.08
T_RS_	1,381	21,414	37.20	33.60	39.90	0.92
TT_S_	1,381	21,413	36.90	33.20	40.00	0.95
RR	1,381	21,360	73.00	12.00	172.00	28.28

^a^T_Vall_: all measures (every 10 min) of vaginal temperatures (°C); T_V4days_: average of the six records per hour corresponding to 08:00, 12:00, 16:00, and 20:00 h during four days (°C); T_ES_: ear skin temperature (°C); T_SS_: shoulder skin temperature (°C); T_RS_: rump skin temperature (°C); T_TS_: tail skin temperature (°C); RR: respiration rate (breaths per minute). Data collection protocols have been described in Johnson et al. [68].

#### Estimation of variance components for traits related to heat stress

As performed for Dataset 1, we also fitted different models including or not non-additive genetic effect of dominance and epistasis to estimate variance components. However, we did not include additive-by-dominance and dominance-by-dominance epistasis effects to avoid overparametrized models, since this second dataset is small.

For the trait HD, the models MAI, MAID, MAIDE1, and MAIE previously mentioned were used. For skin temperatures, vaginal temperatures, PS, and RR repeatability models were fitted:

- MAIpe: $$\textbf{y}=\textbf{X}\varvec{\upbeta }+\textbf{fb}+\textbf{Za}+\textbf{Zpe}+\varvec{\upepsilon }$$;
- MAIDpe: $$\textbf{y}=\textbf{X}\varvec{\upbeta }+\textbf{fb}+\textbf{Za}+\textbf{Zd}+\textbf{Zpe}+\varvec{\upepsilon }$$;
- MAIDEpe: $$\textbf{y}=\textbf{X}\varvec{\upbeta }+\textbf{fb}+\textbf{Za}+\textbf{Zd}+\textbf{Z}{\varvec{e}}_{\varvec{a}\varvec{a}}+\textbf{Zpe}+\varvec{\upepsilon }$$;
- MAIEpe: $$\textbf{y}=\textbf{X}\varvec{\upbeta }+\textbf{fb}+\textbf{Za}+\textbf{Z}{\varvec{e}}_{\varvec{a}\varvec{a}}+\textbf{Zpe}+\varvec{\upepsilon }$$;

where $\textbf{f}$ is the vector of genomic inbreeding coefficient; $\textbf{b}$ is the inbreeding depression; $\varvec{p}\varvec{e} \sim N(0, \varvec{I}{\sigma }_{pe}^{2})$ where ${\sigma }_{pe}^{2}$ is the permanent environmental variance and $\varvec{I}$ an identity matrix; all other effects and matrices in the models have been previously described. The fixed effects for each trait were included as previously defined by Freitas et al. [70]. For T_Vall_, the fixed effects were the concatenation of week and day of measurement; parity; concatenation of barn type and room; and in-barn environmental temperature as a linear covariate. For T_V4days_, the fixed effects were concatenation of week, day, and time of measurement; parity; days in lactation; concatenation of barn type and room; and in-barn environmental temperature as a linear covariate. The fixed effects fitted for skin surface temperature (T_ES_, T_SS_, T_RS_, and T_TS_) and RR were trait recorder; concatenation of week, day, and time of measurement; parity; days in lactation; concatenation of barn type and room; and in-barn environmental temperature as a linear covariate. For PS, the fixed effects were trait recorder; concatenation of week and day of measurement; parity; days in lactation; concatenation of barn type and room; and in-barn environmental temperature as a linear covariate. The fixed effects fitted for HD were trait recorder and parity.

The variance components for dataset 2 were estimated following the same methods described for dataset 1. The analyses were also performed using ASREML software [67]. The estimates of additive heritability (${h}_{a}^{2}$), dominance variance ratio (${h}_{d}^{2}$), and epistatic additive-by-additive variance ratio (${h}_{aa}^{2}$) were computed, as described for dataset 1. The ratio of the total genetic variance ($\widehat{{\sigma }_{g}^{2}}=\widehat{{\sigma }_{a}^{2}}+\widehat{{\sigma }_{d}^{2}}+\widehat{{\sigma }_{aa}^{2}}$) explained by dominance ($\widehat{{\sigma }_{d}^{2}}/\widehat{{\sigma }_{g}^{2}}$) and epistasis additive-by-additive ($\widehat{{\sigma }_{aa}^{2}}/\widehat{{\sigma }_{g}^{2}}$) were also estimated.

#### Model comparison

The AIC [68] and LRT parameters were also used to compare the models’ adjustment. LRT was computed comparing the models including non-additive genetic effects with the additive animal model (MAIpe). The statistic was calculated as previously described and the level of significance used was 0.05.

### Supplementary Information

**Additional file 1: Table S1.** Akaike information criterion (AIC) of model comparison for the purebred pig population.

**Additional file 2: Table S2.** Likelihood ratio test (LRT) of model comparison for the purebred pigs dataset.

**Additional file 3: Table S3.** Akaike information criterion (AIC) of model comparison for the crossbred pig population.

**Additional file 4: Table S4.** Likelihood ratio test (LRT) of model comparison for the crossbred pig dataset.

## Data Availability

The publicly available swine dataset can be downloaded from https://academic.oup.com/g3journal/article/2/4/429/6026060#305071041 [32]. The heat stress dataset can be made available for research purposes through Luiz Brito (britol@purdue.edu).
