# Supplementary material for: Investigating the impact of non-additive genetic effects in the estimation of variance components and genomic predictions for heat tolerance and performance traits in crossbred and purebred pig populations
Source: BMC Genom Data. 2023 Dec 13;24:76. doi: 10.1186/s12863-023-01174-x (PMC10717470; doi:10.1186/s12863-023-01174-x)
Supplement: Supplementary file 1 — Additional file 1: Table S1. Akaike information criterion (AIC) of model comparison for the purebred pig population. [file 12863_2023_1174_MOESM1_ESM.docx]

**Table S1.** Akaike information criterion (AIC) of model comparison for the purebred pig population

| Trait | Model | AIC | Parameters |
| --- | --- | --- | --- |
| T1 | MA | 3,863.83 | 2 |
|  | MAI | 3,863.11 | 2 |
|  | MAE | 3,862.02 | 3 |
|  | MAIE | 3,861.25 | 3 |
|  | MAD | 3,865.83 | 3 |
|  | MAID | 3,865.11 | 3 |
|  | MADE1 | 3,864.02 | 4 |
|  | MAIDE1 | 3,863.25 | 4 |
|  | MADE2 | 3,866.02 | 5 |
|  | MAIDE2 | 3,865.25 | 5 |
|  | MADE3 | 3,868.02 | 6 |
|  | MAIDE3 | 3,867.25 | 6 |
| T2 | MA | 2,712.44 | 2 |
|  | MAI | 2,712.71 | 2 |
|  | MAE | 2,701.22 | 3 |
|  | MAIE | 2,701.36 | 3 |
|  | MAD | 2,713.47 | 3 |
|  | MAID | 2,713.72 | 3 |
|  | MADE1 | 2,702.37 | 4 |
|  | MAIDE1 | 2,702.45 | 4 |
|  | MADE2 | 2,704.37 | 5 |
|  | MAIDE2 | 2,704.45 | 5 |
|  | MADE3 | 2,706.37 | 6 |
|  | MAIDE3 | 2,706.45 | 6 |
| T3 | MA | 2,621.71 | 2 |
|  | MAI | 2,621.59 | 2 |
|  | MAE | 2,580.50 | 3 |
|  | MAIE | 2,580.11 | 3 |
|  | MAD | 2,623.15 | 3 |
|  | MAID | 2,622.99 | 3 |
|  | MADE1 | 2,582.50 | 4 |
|  | MAIDE1 | 2,582.11 | 4 |
|  | MADE2 | 2,570.46 | 5 |
|  | MAIDE2 | 2,570.23 | 5 |
|  | MADE3 | 2,568.15 | 6 |
|  | MAIDE3 | - | - |
| T4 | MA | 7,888.88 | 2 |
|  | MAI | 7,888.00 | 2 |
|  | MAE | 7,888.03 | 3 |
|  | MAIE | 7,887.10 | 3 |
|  | MAD | 7,890.30 | 3 |
|  | MAID | 7,889.40 | 3 |
|  | MADE1 | 7,889.81 | 4 |
|  | MAIDE1 | 7,888.88 | 4 |
|  | MADE2 | 7,891.81 | 5 |
|  | MAIDE2 | 7,890.88 | 5 |
|  | MADE3 | 7,893.81 | 6 |
|  | MAIDE3 | 7,892.88 | 6 |
| T5 | MA | 28,635.05 | 2 |
|  | MAI | 28,624.10 | 2 |
|  | MAE | 28,629.19 | 3 |
|  | MAIE | 28,618.39 | 3 |
|  | MAD | 28,625.71 | 3 |
|  | MAID | 28,616.85 | 3 |
|  | MADE1 | 28,622.47 | 4 |
|  | MAIDE1 | 28,613.47 | 4 |
|  | MADE2 | 28,624.47 | 5 |
|  | MAIDE2 | 28,615.47 | 5 |
|  | MADE3 | 28,626.47 | 6 |
|  | MAIDE3 | 28,617.47 | 6 |
| ^1^MA: $\boldsymbol{y=X\beta+Za+\varepsilon}$ ; MAI: $\boldsymbol{y=X\beta+fb+Za+\varepsilon}$**;** MAE: $\boldsymbol{y=X\beta+Za+Z}\boldsymbol{e}_{\boldsymbol{aa}}\boldsymbol{+\varepsilon}$**;** MAIE: $\boldsymbol{y=X\beta+fb+Za+Z}\boldsymbol{e}_{\boldsymbol{aa}}\boldsymbol{+\varepsilon}$**;** MAD: $\boldsymbol{y=X\beta+Za+Zd+\varepsilon}$**;** MAID: $\boldsymbol{y=X\beta+fb+Za+Zd+\varepsilon}$**;** MADE1: $\boldsymbol{y=X\beta+Za+Zd+Z}\boldsymbol{e}_{\boldsymbol{aa}}\boldsymbol{+\varepsilon}$**;** MAIDE1: $\boldsymbol{y=X\beta+fb+Za+Zd+Z}\boldsymbol{e}_{\boldsymbol{aa}}\boldsymbol{+\varepsilon}$**;** MADE2: $\boldsymbol{y=X\beta+Za+Zd+Z}\boldsymbol{e}_{\boldsymbol{aa}}\mathbf{+Z}\boldsymbol{e}_{\boldsymbol{ad}}\boldsymbol{+\varepsilon}$**;** MAIDE2: $\boldsymbol{y=X\beta+fb+Za+Zd+Z}\boldsymbol{e}_{\boldsymbol{aa}}\mathbf{+Z}\boldsymbol{e}_{\boldsymbol{ad}}\boldsymbol{+\varepsilon}$**;** MADE3: $\boldsymbol{y=X\beta+Za+Zd+Z}\boldsymbol{e}_{\boldsymbol{aa}}\mathbf{+Z}\boldsymbol{e}_{\boldsymbol{ad}}\mathbf{+Z}\boldsymbol{e}_{\boldsymbol{dd}}\boldsymbol{+\varepsilon}$**;** MAIDE3: $\boldsymbol{y=X\beta+fb+Za+Zd+Z}\boldsymbol{e}_{\boldsymbol{aa}}\mathbf{+Z}\boldsymbol{e}_{\boldsymbol{ad}}\mathbf{+Z}\boldsymbol{e}_{\boldsymbol{dd}}\boldsymbol{+\varepsilon}$*Model MAIDE3 did not converge for T3. | | | |
