# Supplementary material for: Investigating the impact of non-additive genetic effects in the estimation of variance components and genomic predictions for heat tolerance and performance traits in crossbred and purebred pig populations
Source: BMC Genom Data. 2023 Dec 13;24:76. doi: 10.1186/s12863-023-01174-x (PMC10717470; doi:10.1186/s12863-023-01174-x)
Supplement: Supplementary file 2 — Additional file 2: Table S2. Likelihood ratio test (LRT) of model comparison for the purebred pigs dataset. [file 12863_2023_1174_MOESM2_ESM.docx]

**Table S2.** Likelihood ratio test (LRT) of model comparison for the purebred pigs dataset.

| Trait | Model^1^ | DF^2^ | LRT | p-value | Model | DF | LRT | p-value |
| --- | --- | --- | --- | --- | --- | --- | --- | --- |
| T1 | MAE | 1 | 3.80 | 0.0513 | MAIE | 1 | 3.88 | 0.0489 |
|  | MAD | 1 | 0.00 | 1.0000 | MAID | 1 | 0.00 | 1.0000 |
|  | MADE1 | 2 | 3.80 | 0.1496 | MAIDE1 | 2 | 3.88 | 0.1437 |
|  | MADE2 | 3 | 3.80 | 0.2839 | MAIDE2 | 3 | 3.88 | 0.2747 |
|  | MADE3 | 4 | 3.80 | 0.4337 | MAIDE3 | 4 | 3.88 | 0.4225 |
| T2 | MAE | 1 | 13.22 | 0.0003 | MAIE | 1 | 13.36 | 0.0003 |
|  | MAD | 1 | 0.98 | 0.3222 | MAID | 1 | 1.00 | 0.3173 |
|  | MADE1 | 2 | 14.08 | 0.0009 | MAIDE1 | 2 | 14.26 | 0.0008 |
|  | MADE2 | 3 | 14.08 | 0.0028 | MAIDE2 | 3 | 14.26 | 0.0026 |
|  | MADE3 | 4 | 14.08 | 0.007 | MAIDE3 | 4 | 14.26 | 0.0065 |
| T3 | MAE | 1 | 43.22 | <0.0001 | MAIE | 1 | 43.48 | <0.0001 |
|  | MAD | 1 | 0.58 | 0.4463 | MAID | 1 | 0.62 | 0.4310 |
|  | MADE1 | 2 | 43.22 | <0.0001 | MAIDE1 | 2 | 43.48 | <0.0001 |
|  | MADE2 | 3 | 57.26 | <0.0001 | MAIDE2 | 3 | 57.36 | <0.0001 |
|  | MADE3 | 4 | 61.56 | <0.0001 | MAIDE3^*^ | 4 | - | - |
| T4 | MAE | 1 | 2.86 | 0.0908 | MAIE | 1 | 2.90 | 0.0886 |
|  | MAD | 1 | 0.58 | 0.4463 | MAID | 1 | 0.60 | 0.4386 |
|  | MADE1 | 2 | 3.06 | 0.2165 | MAIDE1 | 2 | 3.12 | 0.2101 |
|  | MADE2 | 3 | 3.06 | 0.3825 | MAIDE2 | 3 | 3.12 | 0.3735 |
|  | MADE3 | 4 | 3.06 | 0.5478 | MAIDE3 | 4 | 3.12 | 0.5379 |
| T5 | MAE | 1 | 7.88 | 0.0050 | MAIE | 1 | 7.72 | 0.0055 |
|  | MAD | 1 | 11.34 | 0.0008 | MAID | 1 | 9.24 | 0.0024 |
|  | MADE1 | 2 | 16.6 | 0.0002 | MAIDE1 | 2 | 14.64 | 0.0007 |
|  | MADE2 | 3 | 16.6 | 0.0009 | MAIDE2 | 3 | 14.64 | 0.0022 |
|  | MADE3 | 4 | 16.6 | 0.0023 | MAIDE3 | 4 | 14.64 | 0.0055 |
| ^1^MA: $\boldsymbol{y=X\beta+Za+\varepsilon}$ ; MAI: $\boldsymbol{y=X\beta+fb+Za+\varepsilon}$**;** MAE: $\boldsymbol{y=X\beta+Za+Z}\boldsymbol{e}_{\boldsymbol{aa}}\boldsymbol{+\varepsilon}$**;** MAIE: $\boldsymbol{y=X\beta+fb+Za+Z}\boldsymbol{e}_{\boldsymbol{aa}}\boldsymbol{+\varepsilon}$**;** MAD: $\boldsymbol{y=X\beta+Za+Zd+\varepsilon}$**;** MAID: $\boldsymbol{y=X\beta+fb+Za+Zd+\varepsilon}$**;** MADE1: $\boldsymbol{y=X\beta+Za+Zd+Z}\boldsymbol{e}_{\boldsymbol{aa}}\boldsymbol{+\varepsilon}$**;** MAIDE1: $\boldsymbol{y=X\beta+fb+Za+Zd+Z}\boldsymbol{e}_{\boldsymbol{aa}}\boldsymbol{+\varepsilon}$**;** MADE2: $\boldsymbol{y=X\beta+Za+Zd+Z}\boldsymbol{e}_{\boldsymbol{aa}}\mathbf{+Z}\boldsymbol{e}_{\boldsymbol{ad}}\boldsymbol{+\varepsilon}$**;** MAIDE2: $\boldsymbol{y=X\beta+fb+Za+Zd+Z}\boldsymbol{e}_{\boldsymbol{aa}}\mathbf{+Z}\boldsymbol{e}_{\boldsymbol{ad}}\boldsymbol{+\varepsilon}$**;** MADE3: $\boldsymbol{y=X\beta+Za+Zd+Z}\boldsymbol{e}_{\boldsymbol{aa}}\mathbf{+Z}\boldsymbol{e}_{\boldsymbol{ad}}\mathbf{+Z}\boldsymbol{e}_{\boldsymbol{dd}}\boldsymbol{+\varepsilon}$**;** MAIDE3: $\boldsymbol{y=X\beta+fb+Za+Zd+Z}\boldsymbol{e}_{\boldsymbol{aa}}\mathbf{+Z}\boldsymbol{e}_{\boldsymbol{ad}}\mathbf{+Z}\boldsymbol{e}_{\boldsymbol{dd}}\boldsymbol{+\varepsilon}$  ^2^DF: degrees of freedom  *The model MAIDE3 did not converge for T3 | | | | | | | | |
