# Supplementary material for: Investigating the impact of non-additive genetic effects in the estimation of variance components and genomic predictions for heat tolerance and performance traits in crossbred and purebred pig populations
Source: BMC Genom Data. 2023 Dec 13;24:76. doi: 10.1186/s12863-023-01174-x (PMC10717470; doi:10.1186/s12863-023-01174-x)
Supplement: Supplementary file 3 — Additional file 3: Table S3. Akaike information criterion (AIC) of model comparison for the crossbred pig population. [file 12863_2023_1174_MOESM3_ESM.docx]

**Table S3.** Akaike information criterion (AIC) of model comparison for the crossbred pig population

| Trait^1^ | Model^2^ | AIC | Parameters |
| --- | --- | --- | --- |
| TV_all_ | MAIpe | -162156 | 3 |
|  | MAIDpe | -162155 | 4 |
|  | MAIDEpe | -162153 | 5 |
|  | MAIEpe | -162155 | 4 |
| TV_4days_ | MAIpe | -15530.2 | 3 |
|  | MAIDpe | -15528.5 | 4 |
|  | MAIDEpe | -15526.5 | 5 |
|  | MAIEpe | -15528.2 | 4 |
| T_ES_ | MAIpe | 16355.28 | 3 |
|  | MAIDpe | 16357.28 | 4 |
|  | MAIDEpe | 16359.28 | 5 |
|  | MAIEpe | 16357.28 | 4 |
| T_SS_ | MAIpe | 13521.56 | 3 |
|  | MAIDpe | 13523.46 | 4 |
|  | MAIDEpe | 13525.46 | 5 |
|  | MAIEpe | 13523.56 | 4 |
| T_RS_ | MAIpe | 2449.65 | 3 |
|  | MAIDpe | 2451.65 | 4 |
|  | MAIDEpe | 2453.65 | 5 |
|  | MAIEpe | 2451.65 | 4 |
| T_TS_ | MAIpe | 7073.62 | 3 |
|  | MAIDpe | 7075.52 | 4 |
|  | MAIDEpe | 7077.52 | 5 |
|  | MAIEpe | 7075.62 | 3 |
| RR | MAIpe | 149523.2 | 3 |
|  | MAIDpe | 149525.2 | 4 |
|  | MAIDEpe | 149527.2 | 5 |
|  | MAIEpe | 149525.2 | 4 |
| PS | MAIpe | 5593.27 | 3 |
|  | MAIDpe | 5595.22 | 4 |
|  | MAIDEpe | 5588.49 | 5 |
|  | MAIEpe | 5586.49 | 4 |
| HD | MAI | 104.39 | 2 |
|  | MAID | 105.67 | 3 |
|  | MAIDE | 104.7 | 4 |
|  | MAIE | 102.93 | 3 |
| ^1^TV_all_: all measures (every 10 minutes) of vaginal temperatures during four days (°C); TV_4days_: four-time measures of vaginal temperatures during four days (°C); T_ES_: ear skin temperature; T_SS_: shoulder skin temperature; T_RS_: rump skin temperature; T_TS_: tail skin temperature; RR: respiration rate; PS: panting score; HD: hair density.  ^2^MAIpe: $\mathbf{y}\mathbf{=}\boldsymbol{X\beta}\mathbf{+}\mathbf{fb}\mathbf{+}\mathbf{Za}\mathbf{+}\mathbf{Zpe}\mathbf{+}\boldsymbol{\varepsilon}$; MAIEpe: $\mathbf{y}\mathbf{=}\boldsymbol{X\beta}\mathbf{+}\mathbf{fb}\mathbf{+}\mathbf{Za}\mathbf{+}\mathbf{Zpe}\mathbf{+}\mathbf{Z}\boldsymbol{e}_{\boldsymbol{aa}}\mathbf{+}\boldsymbol{\varepsilon}$; MAIDpe: $\mathbf{y}\mathbf{=}\boldsymbol{X\beta}\mathbf{+}\mathbf{fb}\mathbf{+}\mathbf{Za}\mathbf{+}\mathbf{Zpe}\mathbf{+}\mathbf{Zd}\mathbf{+}\boldsymbol{\varepsilon}$; MAIDEpe: $\mathbf{y}\mathbf{=}\boldsymbol{X\beta}\mathbf{+}\mathbf{fb}\mathbf{+}\mathbf{Za}\mathbf{+}\mathbf{Zd}\mathbf{+}\mathbf{Z}\boldsymbol{e}_{\boldsymbol{aa}}\mathbf{+}\mathbf{Zpe}\mathbf{+}\boldsymbol{\varepsilon}$; MAI: $\mathbf{y}\mathbf{=}\boldsymbol{X\beta}\mathbf{+}\mathbf{fb}\mathbf{+}\mathbf{Za}\mathbf{+}\boldsymbol{\varepsilon}$; MAIE: $\mathbf{y}\mathbf{=}\boldsymbol{X\beta}\mathbf{+}\mathbf{fb}\mathbf{+}\mathbf{Za}\mathbf{+}\mathbf{Z}\boldsymbol{e}_{\boldsymbol{aa}}\mathbf{+}\boldsymbol{\varepsilon}$; MAID: $\mathbf{y}\mathbf{=}\boldsymbol{X\beta}\mathbf{+}\mathbf{fb}\mathbf{+}\mathbf{Za}\mathbf{+}\mathbf{Zd}\mathbf{+}\boldsymbol{\varepsilon}$; MAIDE1: $\mathbf{y}\mathbf{=}\boldsymbol{X\beta}\mathbf{+}\mathbf{fb}\mathbf{+}\mathbf{Za}\mathbf{+}\mathbf{Zd}\mathbf{+}\mathbf{Z}\boldsymbol{e}_{\boldsymbol{aa}}\mathbf{+}\boldsymbol{\varepsilon}$ | | | |
