# Supplementary material for: Investigating the impact of non-additive genetic effects in the estimation of variance components and genomic predictions for heat tolerance and performance traits in crossbred and purebred pig populations
Source: BMC Genom Data. 2023 Dec 13;24:76. doi: 10.1186/s12863-023-01174-x (PMC10717470; doi:10.1186/s12863-023-01174-x)
Supplement: Supplementary file 4 — Additional file 4: Table S4. Likelihood ratio test (LRT) of model comparison for the crossbred pig dataset. [file 12863_2023_1174_MOESM4_ESM.docx]

**Table S4.** Likelihood ratio test (LRT) of model comparison for the crossbred pig dataset.

| Trait^1^ | Model^2^ | DF^3^ | LRT | p-value |
| --- | --- | --- | --- | --- |
| TV_all_ | MAIDpe | 1 | 0.4 | 0.5271 |
|  | MAIDEpe | 2 | 0.8 | 0.6703 |
|  | MAIEpe | 1 | 0.6 | 0.4386 |
| TV_4days_ | MAIDpe | 1 | 0.34 | 0.5598 |
|  | MAIDEpe | 2 | 0.34 | 0.8437 |
|  | MAIEpe | 1 | 0 | 1.0000 |
| T_ES_ | MAIDpe | 1 | 0 | 1.0000 |
|  | MAIDEpe | 2 | 0 | 1.0000 |
|  | MAIEpe | 1 | 0 | 1.0000 |
| T_SS_ | MAIDpe | 1 | 0.1 | 0.7518 |
|  | MAIDEpe | 2 | 0.1 | 0.9512 |
|  | MAIEpe | 1 | 0 | 1.0000 |
| T_RS_ | MAIDpe | 1 | 0 | 1.0000 |
|  | MAIDEpe | 2 | 0 | 1.0000 |
|  | MAIEpe | 1 | 0 | 1.0000 |
| T_TS_ | MAIDpe | 1 | 0.1 | 0.7518 |
|  | MAIDEpe | 2 | 0.1 | 0.9512 |
|  | MAIEpe | 1 | 0 | 1.0000 |
| RR | MAIDpe | 1 | 0 | 1.0000 |
|  | MAIDEpe | 2 | 0 | 1.0000 |
|  | MAIEpe | 1 | 0 | 1.0000 |
| PS | MAIDpe | 1 | 0.04 | 0.8415 |
|  | MAIDEpe | 2 | 8.76 | 0.0125 |
|  | MAIEpe | 1 | 8.76 | 0.0031 |
| HD | MAID | 1 | 0.7194 | 0.3963 |
|  | MAIDE | 2 | 3.6876 | 0.1582 |
|  | MAIE | 1 | 3.4596 | 0.0629 |
| ^1^TV_all_: all measures (every 10 minutes) of vaginal temperatures during four days (°C); TV_4days_: four-time measures of vaginal temperatures during four days (°C); T_ES_: ear skin temperature; T_SS_: shoulder skin temperature; T_RS_: rump skin temperature; T_TS_: tail skin temperature; RR: respiration rate; PS: panting score; HD: hair density.  ^2^MAIpe: $\mathbf{y}\mathbf{=}\boldsymbol{X\beta}\mathbf{+}\mathbf{fb}\mathbf{+}\mathbf{Za}\mathbf{+}\mathbf{Zpe}\mathbf{+}\boldsymbol{\varepsilon}$; MAIEpe: $\mathbf{y}\mathbf{=}\boldsymbol{X\beta}\mathbf{+}\mathbf{fb}\mathbf{+}\mathbf{Za}\mathbf{+}\mathbf{Zpe}\mathbf{+}\mathbf{Z}\boldsymbol{e}_{\boldsymbol{aa}}\mathbf{+}\boldsymbol{\varepsilon}$; MAIDpe: $\mathbf{y}\mathbf{=}\boldsymbol{X\beta}\mathbf{+}\mathbf{fb}\mathbf{+}\mathbf{Za}\mathbf{+}\mathbf{Zpe}\mathbf{+}\mathbf{Zd}\mathbf{+}\boldsymbol{\varepsilon}$; MAIDEpe: $\mathbf{y}\mathbf{=}\boldsymbol{X\beta}\mathbf{+}\mathbf{fb}\mathbf{+}\mathbf{Za}\mathbf{+}\mathbf{Zd}\mathbf{+}\mathbf{Z}\boldsymbol{e}_{\boldsymbol{aa}}\mathbf{+}\mathbf{Zpe}\mathbf{+}\boldsymbol{\varepsilon}$; MAI: $\mathbf{y}\mathbf{=}\boldsymbol{X\beta}\mathbf{+}\mathbf{fb}\mathbf{+}\mathbf{Za}\mathbf{+}\boldsymbol{\varepsilon}$; MAIE: $\mathbf{y}\mathbf{=}\boldsymbol{X\beta}\mathbf{+}\mathbf{fb}\mathbf{+}\mathbf{Za}\mathbf{+}\mathbf{Z}\boldsymbol{e}_{\boldsymbol{aa}}\mathbf{+}\boldsymbol{\varepsilon}$; MAID: $\mathbf{y}\mathbf{=}\boldsymbol{X\beta}\mathbf{+}\mathbf{fb}\mathbf{+}\mathbf{Za}\mathbf{+}\mathbf{Zd}\mathbf{+}\boldsymbol{\varepsilon}$; MAIDE1: $\mathbf{y}\mathbf{=}\boldsymbol{X\beta}\mathbf{+}\mathbf{fb}\mathbf{+}\mathbf{Za}\mathbf{+}\mathbf{Zd}\mathbf{+}\mathbf{Z}\boldsymbol{e}_{\boldsymbol{aa}}\mathbf{+}\boldsymbol{\varepsilon}$  ^3^DF: degrees of freedom | | | | |
